# Supplementary material for: Who with whom: functional coordination of E2 enzymes by RING E3 ligases during poly‐ubiquitylation
Source: EMBO J. 2020 Oct 5;39(22):e104863. doi: 10.15252/embj.2020104863 (PMC7667886; doi:10.15252/embj.2020104863)
Supplement: Supplementary file 1 — Appendix [file EMBJ-39-e104863-s001.pdf]

Appendix

Who with whom: Functional coordination of E2 enzymes by RING E3 ligases during poly-ubiquitylation

Appendix Figure S1	Protein degradation assays for the Hrd1 model substrate PrA*-3xHA
Appendix Figure S2	Protein degradation assays for the Doa10 model substrate Deg1-eGFP$_2$
Appendix Figure S3	NMR analysis of the His$_6$-Ubc13-SS-(^{15}N)Ub conjugates
Appendix Figure S4	Mapping of conjugate CSPs onto Ub structure
Appendix Figure S5	Validation of yeast strains

Appendix Table S1	Bacterial Expression Plasmids
Appendix Table S2	Yeast Expression Plasmids
Appendix Table S3	Yeast Strains
Appendix Table S4	Antibodies

PrA*-3xHA degradation (Hrd1 substrate)

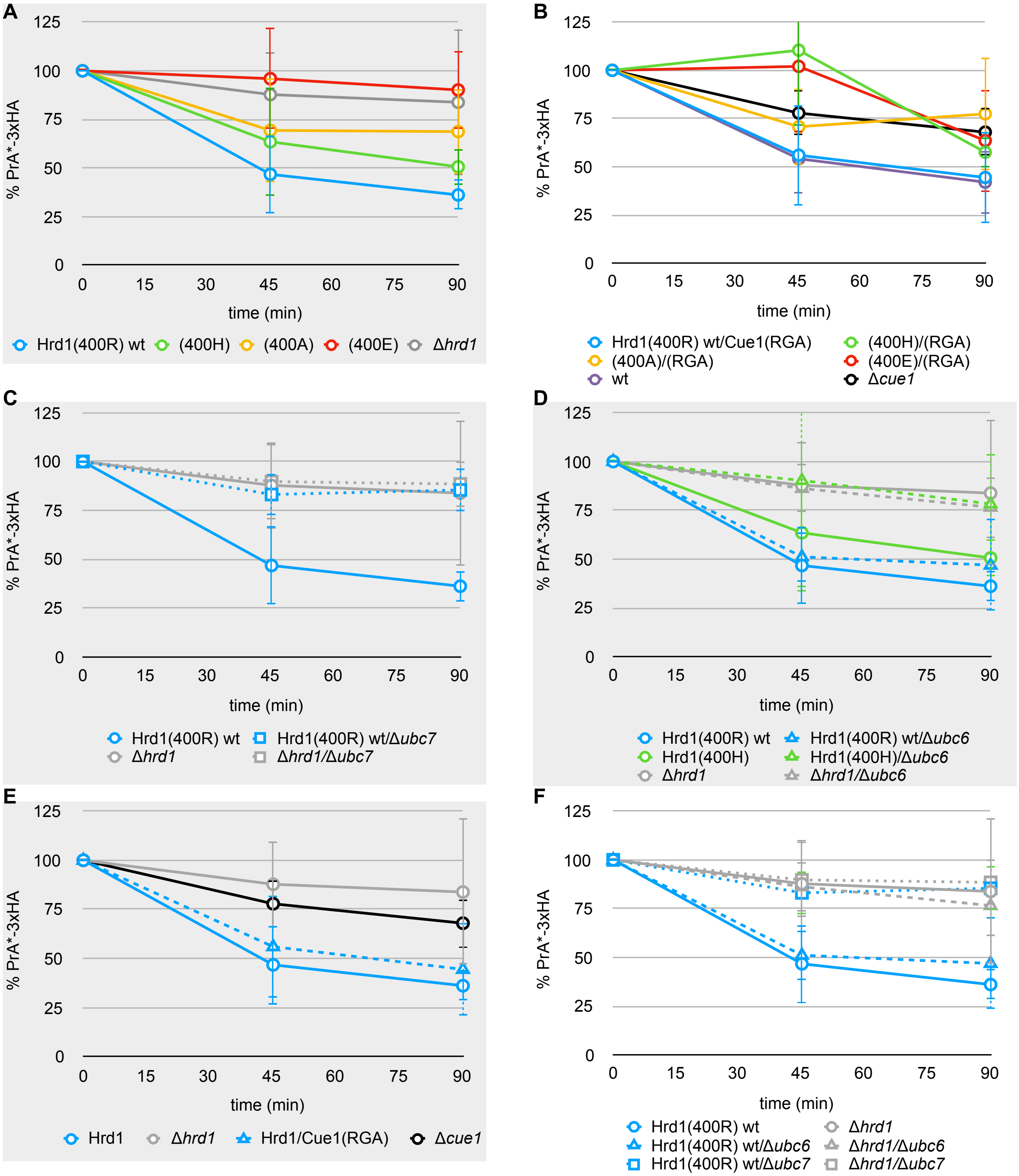

Appendix Figure S1. Protein degradation assays for the Hrd1 model substrate PrA*-3xHA.

A – D Protein degradation in indicated yeast strains monitored by pulse-chase experiments for the Hrd1 model substrate PrA*-3xHA. Values for each time point are reported as means ± standard deviation (n = 4).

E + F Data shown in A and B or in C and D, respectively, are shown in rearranged organization for clarity.

Grey background indicates data already shown in main figures (Fig. 4A, 6A, 6D and 3D); for better comparison, all assays are shown here side-by-side.

Deg1-eGFP$_2$ degradation (Doa10 substrate)

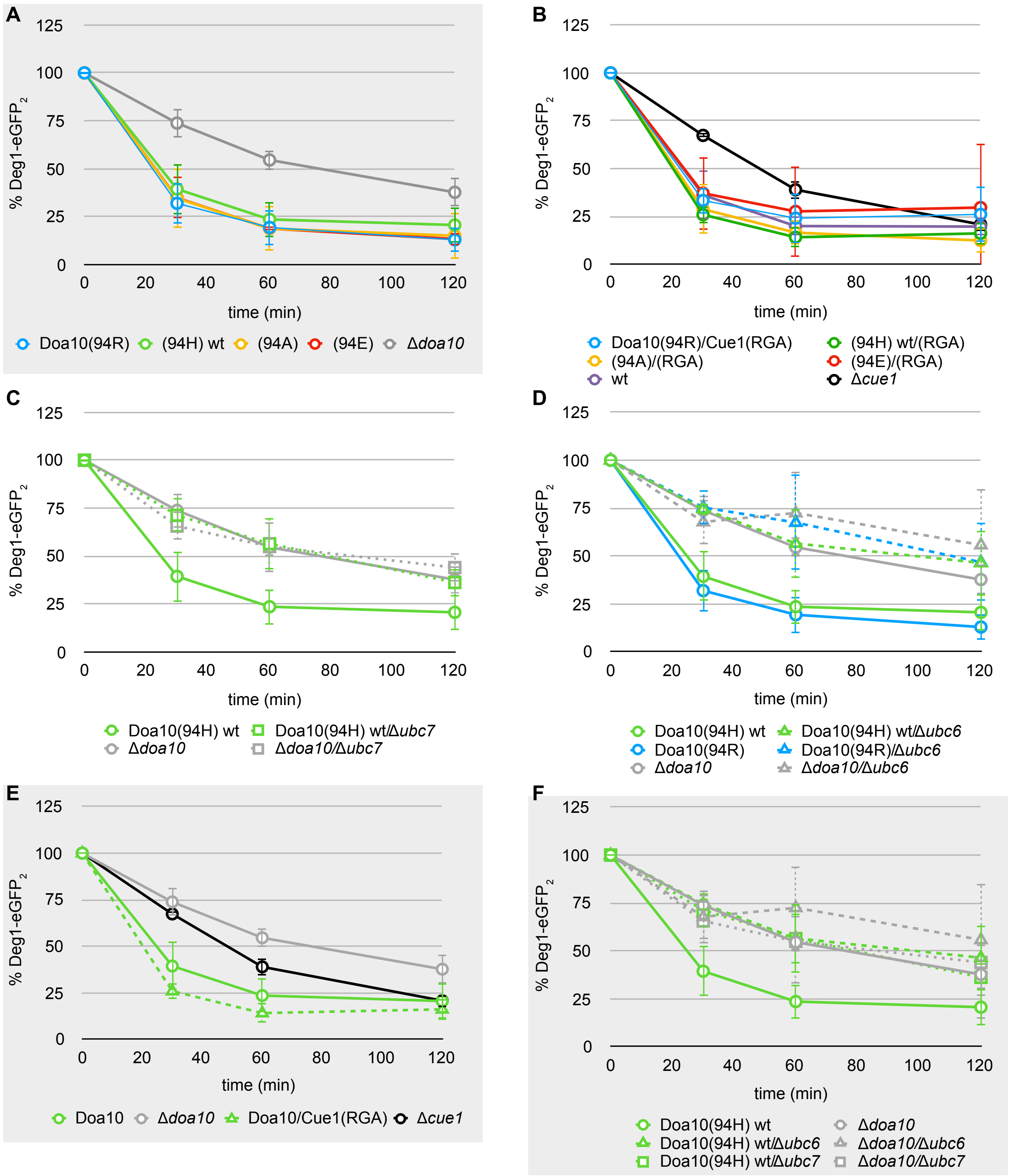

Appendix Figure S2. Protein degradation assays for the Doa10 model substrate Deg1-eGFP$_2$.

A – D Protein degradation in indicated yeast strains monitored by CHX decay assays for the Doa10 model substrate Deg1-eGFP$_2$. Values for each time point are reported as means ± standard deviation (n = 3).

E + F Data shown in A and B or in C and D, respectively, are shown in rearranged organization for clarity.

Grey background indicates data already shown in main figures (Fig. 4A, 3D and 6B); for better comparison, all assays are shown here side-by-side.

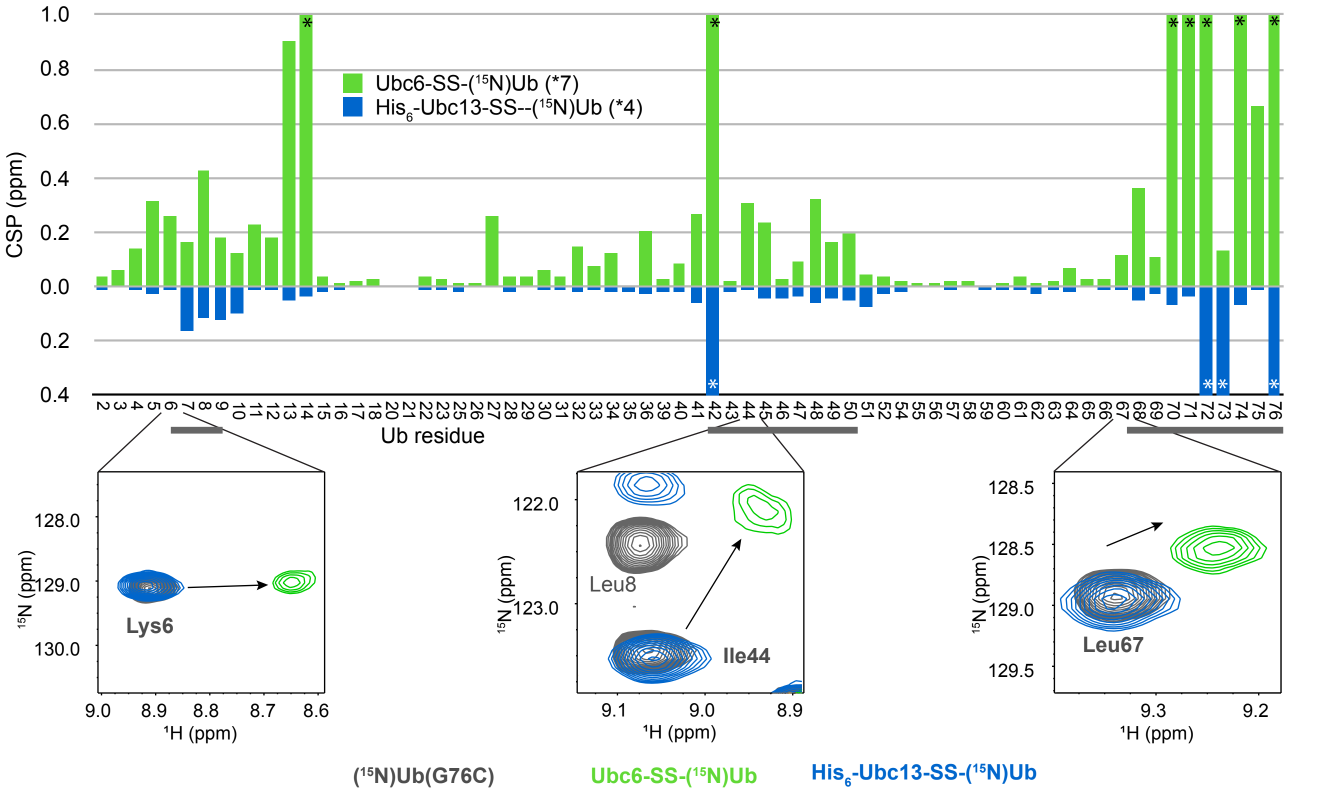

Appendix Figure S3. NMR analysis of the His_6-Ubc13-SS-(^{15}N)Ub conjugates.
CSPs of (^{15}N)Ub(G76C) conjugated to indicated E2s via disulfide bond (775 μM each) compared to free (^{15}N)Ub(G76C) are shown; Ub resides known to be involved in the closed conformation interface are underlined in grey; * = number of resonances with perturbations too large to be assigned confidently (shown as off-scale in diagram). Bottom: Spectra of resonances for selected residues in Ub's hydrophobic patch for free Ub(G76C) (gray) and Ub conjugated to Ubc6 (green) and His_6-Ubc13 (blue).

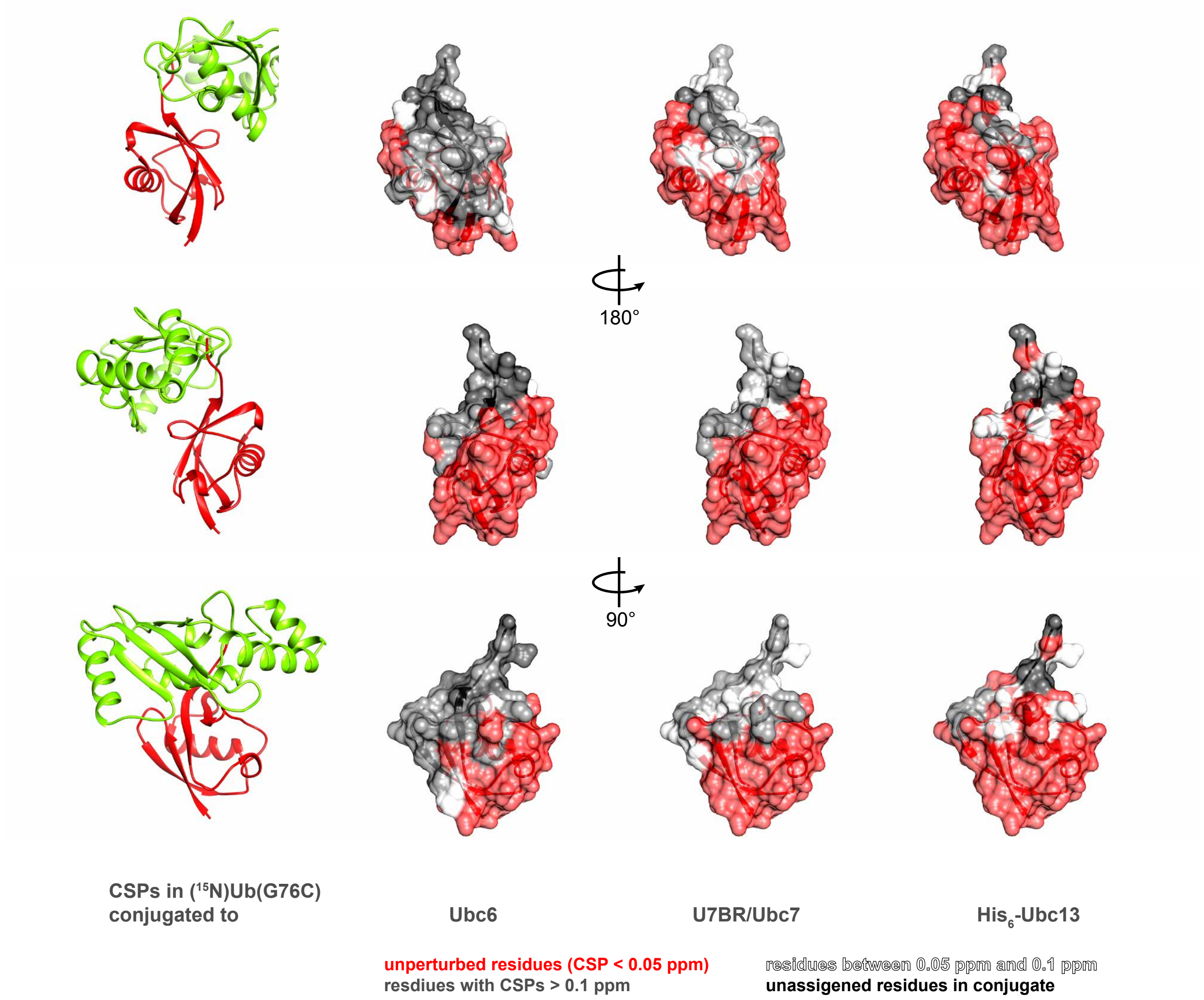

Appendix Figure S4. Mapping of conjugate CSPs on Ub structure.
CSPs of conjugated Ub from Fig. 5B and S3 are mapped onto the Ub structure. Left: structure of UbcH5a-Ub conjugate in the closed conformation, with the E2 shown in green and Ub shown in red (PDB: 4AP4). Next to that from left to right: structure of Ub (without E2 in same positions) with CSPs for Ub in the Ubc6, U7BR/Ubc7 and His_6-Ubc13 conjugate. Unperturbed residues are shown in red. Residues with CSPs between 0.05 and 0.1 ppm are represented in white, residues with CSPs larger than 0.1 ppm in grey and residues lost in the conjugate in black.

A

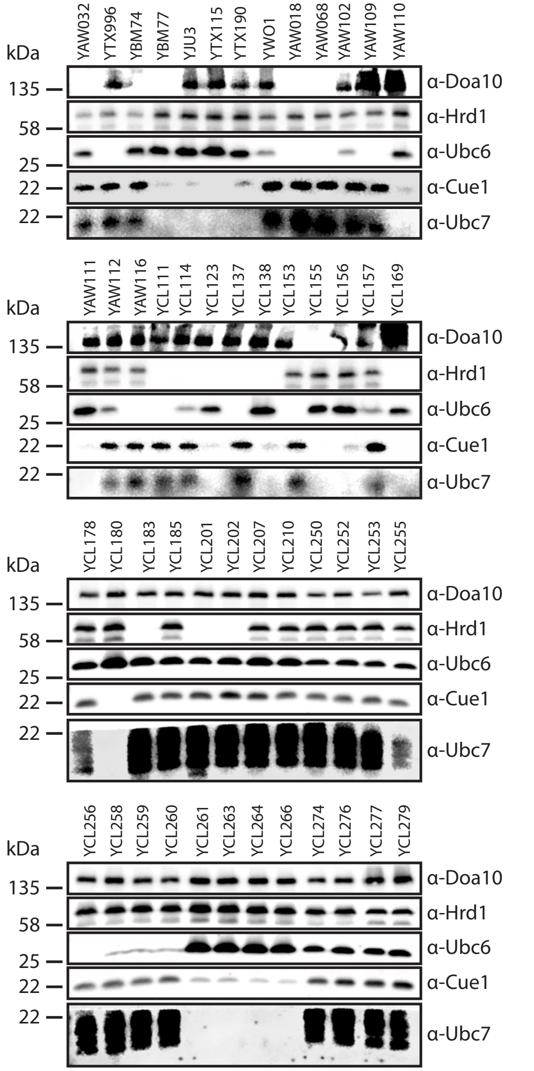

B

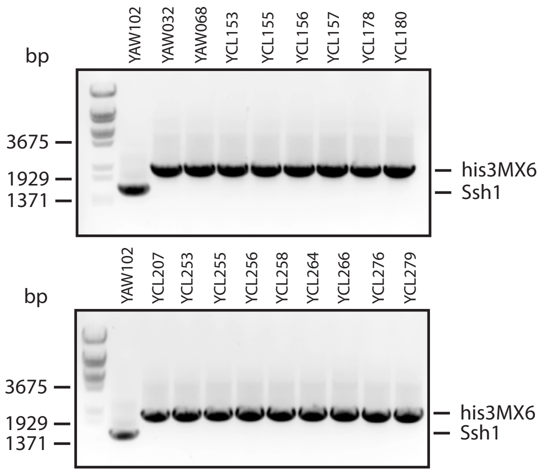

Appendix Figure S5. Validation of yeast strains.
Validation of indicated yeast strains used in this study on the protein level via indicated immunoblots A and on the DNA level by PCR analyzed on agarose gels B.

Appendix Table S1. Bacterial Expression Plasmids

insert	vector	used in	internal #	reference
GST-Cue1-His_6 (aa24-203)	pGEX-6p1	substrate ubiquitylation assay, chain elongation assay	pTX410	(Bagola et al., 2013)
GST-Cue1(LAP76-78RGA)-His_6 (24-203)	pGEX-6p1	chain elongation assay	pTX411	(Bagola et al., 2013)
GST-Ubc7 (aa2-165)	pGEX-6p1	discharge assay, substrate ubiquitylation assay, chain elongation assay	pTX249	(Bagola et al., 2013)
Uba1-His_6	pET32	substrate ubiquitylation assay, chain elongation assay	pTX481	(Berndsen and Wolberger, 2011)
GST-Cdc34	pGEX-6p1	Synthesis of K48-linked Ub chains	pMD26	(Delbrück et al., 2016)
hUb-His_6	pETM60	chain elongation assay	pMD11	(Delbrück et al., 2016)
hUb(S20C)	pETM60	chain elongation assay	pMD12	(Delbrück et al., 2016)
GST-Ubc6 (aa 2-230)	pGEX-6p1	substrate ubiquitylation assay, chain elongation assay	pAW039	(Weber et al., 2016)
His_6-hUbc13	pET24	NMR	REK8	(Pruneda et al., 2011)
His_6-Ulp1	pET28b	purification of SUMO-tagged proteins		Addgene
GST-hSENP1 (aa 419-644)	pGEX-4T1	purification of SUMO-tagged proteins	REK9	(Bossis and Melchior, 2006)
hUb	pET15b	discharge assay	REK1	(Christensen et al., 2007)
Uba1-His_6	pET21d	discharge assay	REK4	(Christensen et al., 2007)
hUb	pETM60	substrate ubiquitylation assay, chain elongation assay	pMD10	(Rogov et al., 2012)
GST-Doa10 (aa 19-102)	pGEX-6p1	NMR, discharge assay, chain elongation assay	pToR3	this study
GST-Doa10(H94A) (aa 19-102)	pGEX-6p1	NMR, discharge assay, chain elongation assay	pToR5	this study
GST-Doa10(H94E) (aa 19-102)	pGEX-6p1	discharge assay, chain elongation assay	pToR6	this study
GST-Doa10(H94R) (aa 19-102)	pGEX-6p1	NMR, discharge assay, chain elongation assay	pToR4	this study
GST-RNAse A-TUB (aa 42-150)	pGEX-6p1	substrate ubiquitylation assay	pCL172	this study
GST-S-Doa10 (aa 19-102)	pGEX-6p1	substrate ubiquitylation assay	pCL167	this study
GST-S-Doa10(H94A) (aa 19-102)	pGEX-6p1	substrate ubiquitylation assay	pCL177	this study
GST-S-Doa10(H94E) (aa 19-102)	pGEX-6p1	substrate ubiquitylation assay	pCL178	this study
GST-S-Doa10(H94R) (aa 19-102)	pGEX-6p1	substrate ubiquitylation assay	pCL176	this study
GST-Ubc6 (aa 2-179)	pGEX-6p1	NMR, discharge assay	REK5	this study
His_6-hSUMO3-Hrd1 (aa 325-412)	pET28a (Meul-meester et al., 2008)	NMR, discharge assay, chain elongation assay	pToR1	this study
His_6-hSUMO3-Hrd1(R400A) (aa 325-412)	pET28a (Meul-meester et al., 2008)	discharge assay, chain elongation assay	pCL153	this study
His_6-hSUMO3-Hrd1(R400E) (aa 325-412)	pET28a (Meul-meester et al., 2008)	discharge assay, chain elongation assay	pCL151	this study
His_6-hSUMO3-Hrd1(R400H) (aa 325-412)	pET28a (Meul-meester et al., 2008)	NMR, discharge assay, chain elongation assay	pCL160	this study
His_6-hSUMO3-S-Hrd1 (aa 325-412)	pET28a (Meul-meester et al., 2008)	substrate ubiquitylation assay	pCL166	this study
His_6-hSUMO3-S-Hrd1(R400H) (aa 325-412)	pET28a (Meul-meester et al., 2008)	substrate ubiquitylation assay	pCL173	this study
His_6-hSUMO3-U7BR-His_6 (aa 150-203)	pET28a (Meul-meester et al., 2008)	NMR, discharge assays	REK6	this study
His_6-hSUMO3-Ubc7(C89only) (aa 2-165) C89only = C93A/C141S	pET28a (Meul-meester et al., 2008)	NMR	REK7	this study
His_6-SUMO3-S-Hrd1(R400A) (aa 325-412)	pET28a (Meul-meester et al., 2008)	substrate ubiquitylation assay	pCL174	this study
His_6-SUMO3-S-Hrd1(R400E) (aa 325-412)	pET28a (Meul-meester et al., 2008)	substrate ubiquitylation assay	pCL175	this study
hUb(G76C)	pET15b	NMR	REK2	this study
hUb(K48R)	pET15b	discharge assay, substrate ubiquitylation assay	REK3	this study

Appendix Table S2. Yeast Expression Plasmids

insert	vector	internal #	reference
CPY*_K0-HA	pRS315	pRB256	(Baldridge and Rapoport, 2016)
FLAG-Sbh2	pRS414	pTR1646	(Weber et al., 2016)
Deg1-eGFP$_2$	pRS414	pUL038	(Lenk and Sommer, 2000)
Hrd1(R400H)	pRS416	pAW183	this study
Hrd1	pRS416	pCL083	this study
Hrd1(R400A)	pRS416	pCL084	this study
Hrd1	pRS317	pCL142	this study
Hrd1(R400A)	pRS317	pCL143	this study
PrA*-3xHA	pRS317	pCL149	this study
Hrd1(R400E)	pRS317	pCL152	this study
Hrd1(R400E)	pRS416	pCL157	this study
Hrd1(R400H)	pRS317	pCL161	this study

Appendix Table S3. Yeast Strains

strain #	genotype	reference
YAW032	*Δdoa10::kanMX6, Δssh1::HIS3, MATa*	(Weber et al., 2016)
YTX996	*Δubc6::HIS3, MATα*	(Weber et al., 2016)
YBM74	*Δdoa10::kanMX6, MATα*	Laboratory of Prof. Sommer
YBM77	*Δdoa10::kanMX6, Δubc7::LEU2, MATα*	Laboratory of Prof. Sommer
YJU3	*Ubc7(C89S), MATa*	Laboratory of Prof. Sommer
YTX115	*Δcue1::LEU2, MATα*	Laboratory of Prof. Sommer
YTX190	*Δubc7::LEU2, MATα*	Laboratory of Prof. Sommer
YWO1	*trp1-1 (am), his-Δ200, ura3-52, lys2-801, leu2-3, -112, MATα*	(Seufert and Jentsch, 1990)
YAW018	*Δdoa10::kanMX6, Δubc6::HIS3, MATα*	this study
YAW068	*Δdoa10::kanMX6, Δssh1::HIS3, Δubc6::LEU2, MATa*	this study
YAW102	*Doa10 H94E, MATα*	this study
YAW109	*Doa10 H94E, Δubc6::HIS3, MATα*	this study
YAW110	*Doa10 H94E, Δubc7::LEU2, MATα*	this study
YAW111	*Doa10 H94E, Δssh1::HIS3, Δubc7::LEU2, MATα*	this study
YAW112	*Doa10 H94E, Δssh1::HIS3, MATa*	this study
YAW116	*Doa10 H94E, Δssh1::HIS3, Δubc6::HIS3, MATα*	this study
YCL111	*Δhrd1::TRP1, Δubc6::HIS3, MATa*	this study
YCL114	*Δhrd1::TRP1, MATα*	this study
YCL123	*Δhrd1::TRP1, Δubc7::LEU2, MATa*	this study
YCL137	*Δhrd1::TRP1, Δubc6::HIS3, 6xMyc-Hmg2::URA3, MATa*	this study
YCL138	*Δhrd1::TRP1, Δubc7::LEU2, 6xMyc-Hmg2::URA3, MATa*	this study
YCL153	*Δssh1::HIS3, Δubc6::HIS3, MATα*	this study
YCL155	*Δdoa10::kanMX6, Δssh1::HIS3, Δubc7::LEU2, MATa*	this study
YCL156	*Δssh1::HIS3, Δubc7::LEU2, MATa*	this study
YCL157	*Δssh1::HIS3, MATa*	this study
YCL169	*Δhrd1::TRP1, Δcue1::LEU2, MATα*	this study

Appendix Table S3. Yeast Strains - continued

strain #	genotype	reference
YCL178	*Δssh1::HIS3, Cue1 LAP(76-78)RGA, MATa*	this study
YCL180	*Δssh1::HIS3, Δcue1::LEU2, MATa*	this study
YCL183	*Δhrd1::TRP1, Cue1 LAP(76-78)RGA, MATa*	this study
YCL185	*Cue1 LAP(76-78)RGA, MATα*	this study
YCL201	*Δhrd1::TRP1, 6xMyc-Hmg2::URA3, MATα*	this study
YCL202	*Δhrd1::TRP1, Cue1 LAP(76-78)RGA, 6xMyc-Hmg2::URA3, MATα*	this study
YCL207	*Doa10 H94E, Δssh1::HIS3, Cue1 LAP(76-78)RGA, MATa*	this study
YCL210	*Doa10 H94E, Cue1 LAP(76-78)RGA, MATa*	this study
YCL250	*Doa10 H94R, MATa*	this study
YCL252	*Doa10 H94A, MATa*	this study
YCL253	*Doa10 H94R, Δssh1::HIS3, MATα*	this study
YCL255	*Doa10 H94A, Δssh1::HIS3, MATα*	this study
YCL256	*Doa10 H94R, Δssh1::HIS3, Δubc6::HIS3, MATα*	this study
YCL258	*Doa10 H94A, Δssh1::HIS3, Δubc6::HIS3, MATa*	this study
YCL259	*Doa10 H94R, Δubc6::HIS3, MATα*	this study
YCL260	*Doa10 H94A, Δubc6::HIS3, MATα*	this study
YCL261	*Doa10 H94R, Δubc7::LEU2, MATα*	this study
YCL263	*Doa10 H94A, Δubc7::LEU2, MATα*	this study
YCL264	*Doa10 H94R, Δssh1::HIS3, Δubc7::LEU2, MATα*	this study
YCL266	*Doa10 H94A, Δssh1::HIS3, Δubc7::LEU2, MATa*	this study
YCL274	*Doa10 H94R, Cue1 LAP(76-78)RGA, MATα*	this study
YCL276	*Doa10 H94R, Δssh1::HIS3, Cue1 LAP(76-78)RGA, MATα*	this study
YCL277	*Doa10 H94A, Cue1 LAP(76-78)RGA, MATα*	this study
YCL279	*Doa10 H94A, Δssh1::HIS3, Cue1 LAP(76-78)RGA, MATα*	this study

Appendix Table S4. Antibodies

primary antibody	dilution	source
Monoclonal α-FLAG (m)	1:2,000	Sigma-Aldrich F3165
Monoclonal α-GFP (m)	1:1,000	Living Colors 632375
Monoclonal α-HA (m)	1:5,000	Sigma-Aldrich H9658
Monoclonal α-Myc (m)	1:2,000	Sigma-Aldrich M5546
Polyclonal α-Cdc48 (rb)	1:10,000	(Neuber et al., 2005)
Polyclonal α-Cue1 (rb)	1:1,000	(Biederer et al., 1997)
Polyclonal α-Doa10 (rb)	1:10,000	(Bagola et al., 2013)
Polyclonal α-Hrd1 (rb)	1:10,000	(Horn et al., 2009)
Polyclonal α-RNase1 (rb)	1:1,000	abcam ab94417
Polyclonal α-Ubc6 (rb)	1:5,000	(Weber et al., 2016)
Polyclonal α-Ubc7 (rb)	1:1,000	(Neuber et al., 2005)
**secondary antibody**	**dilution**	**source**
IRDye® 680RD α-m IgG (g)	1:10,000	LI-COR 926-68070
IRDye® 800CW α-rb IgG (g)	1:10,000	LI-COR 926-32211
Polyclonal α-rb IgG (g), HRP-conjugated	1:10,000	Sigma A0545

References - Appendix

Bagola, K., Delbrück, von, M., Dittmar, G., Scheffner, M., Ziv, I., Glickman, M.H., Ciechanover, A., Sommer, T., 2013. Ubiquitin binding by a CUE domain regulates ubiquitin chain formation by ERAD E3 ligases. Molecular Cell 50, 528–539. doi:10.1016/j.molcel.2013.04.005

Baldridge, R.D., Rapoport, T.A., 2016. Autoubiquitination of the Hrd1 Ligase Triggers Protein Retrotranslocation in ERAD. Cell 166, 394–407. doi:10.1016/j.cell.2016.05.048

Berndsen, C.E., Wolberger, C., 2011. A spectrophotometric assay for conjugation of ubiquitin and ubiquitin-like proteins. Analytical Biochemistry 418, 102–110. doi:10.1016/j.ab.2011.06.034

Biederer, T., Volkwein, C., Sommer, T., 1997. Role of Cue1p in ubiquitination and degradation at the ER surface. Science 278, 1806–1809. doi:10.1126/science.278.5344.1806

Bossis, G., Melchior, F., 2006. Regulation of SUMOylation by reversible oxidation of SUMO conjugating enzymes. Molecular Cell 21, 349–357. doi:10.1016/j.molcel.2005.12.019

Christensen, D.E., Brzovic, P.S., Klevit, R.E., 2007. E2-BRCA1 RING interactions dictate synthesis of mono- or specific polyubiquitin chain linkages. Nat. Struct. Mol. Biol. 14, 941–948. doi:10.1038/nsmb1295

Delbrück, von, M., Kniss, A., Rogov, V.V., Pluska, L., Bagola, K., Löhr, F., Güntert, P., Sommer, T., Dötsch, V., 2016. The CUE Domain of Cue1 Aligns Growing Ubiquitin Chains with Ubc7 for Rapid Elongation. Molecular Cell 62, 918–928. doi:10.1016/j.molcel.2016.04.031

Horn, S.C., Hanna, J., Hirsch, C., Volkwein, C., SchUtz, A., Heinemann, U., Sommer, T., Jarosch, E., 2009. Usa1 Functions as a Scaffold of the HRD-Ubiquitin Ligase. Molecular Cell 36, 782–793. doi:10.1016/j.molcel.2009.10.015

Lenk, U., Sommer, T., 2000. Ubiquitin-mediated Proteolysis of a Short-lived Regulatory Protein Depends on Its Cellular Localization. Journal of Biological Chemistry 275, 39403–39410. doi:10.1074/jbc.274.40.28708

Meulmeester, E., Kunze, M., Hsiao, H.H., Urlaub, H., Melchior, F., 2008. Mechanism and consequences for paralog-specific sumoylation of ubiquitin-specific protease 25. Molecular Cell 30, 610–619. doi:10.1016/j.molcel.2008.03.021

Neuber, O., Jarosch, E., Volkwein, C., Walter, J., Sommer, T., 2005. Ubx2 links the Cdc48 complex to ER-associated protein degradation. Nat Cell Biol 7, 993–998. doi:10.1002/(SICI)1097-0061(199907)15:10B<963::AID-YEA399>3.0.CO;2-W

Pruneda, J.N., Stoll, K.E., Bolton, L.J., Brzovic, P.S., Klevit, R.E., 2011. Ubiquitin in Motion: Structural Studies of the Ubiquitin-Conjugating Enzyme-Ubiquitin Conjugate. Biochemistry 50, 1624–1633. doi:10.1021/bi101913m

Rogov, V.V., Rozenknop, A., Rogova, N.Y., Löhr, F., Tikole, S., Jaravine, V., Güntert, P., Dikic, I., Dötsch, V., 2012. A Universal Expression Tag for Structural and Functional Studies of Proteins. Chembiochem 13, 959–963. doi: 10.1074/jbc.M802182200

Seufert, W., Jentsch, S., 1990. Ubiquitin-conjugating enzymes UBC4 and UBC5 mediate selective degradation of short-lived and abnormal proteins. The EMBO Journal 9, 543–550. doi:10.1002/j.1460-2075.1990.tb08141.x

Weber, A., Cohen, I., Popp, O., Dittmar, G., Reiss, Y., Sommer, T., Ravid, T., Jarosch, E., 2016. Sequential Poly-ubiquitylation by Specialized Conjugating Enzymes Expands the Versatility of a Quality Control Ubiquitin Ligase. Molecular Cell 63, 827–839. doi:10.1016/j.molcel.2016.07.020
