# Supplementary material for: Who with whom: functional coordination of E2 enzymes by RING E3 ligases during poly‐ubiquitylation
Source: EMBO J. 2020 Oct 5;39(22):e104863. doi: 10.15252/embj.2020104863 (PMC7667886; doi:10.15252/embj.2020104863)
Supplement: Supplementary file 2 — Expanded View Figures PDF [file EMBJ-39-e104863-s002.pdf]

Expanded View Figures

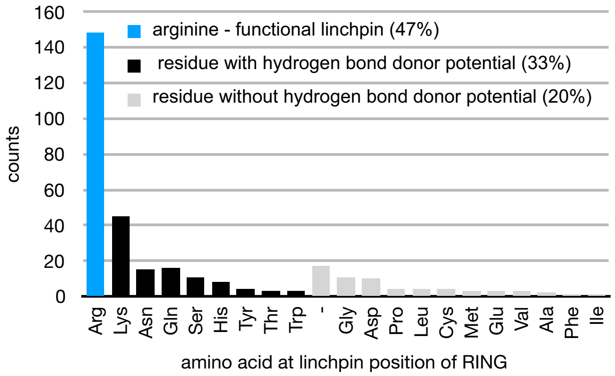

Figure EV1. Amino acid frequencies at the linchpin position of all human RING domains.

Histogram of amino acids at the linchpin position of all 316 human RING domains. The linchpin position was defined as the residue at the $n + 1$ position after the final Zn^{2+} ligand coordinating residues. "–" refers to RING domains that feature a gap at the linchpin position in the multiple sequence alignment performed for all RING domains. SP-RING domains of the PIAS SUMO E3 ligases and RING1 domains of all human RBR E3 ligases were excluded from this analysis.

Figure EV2. *In vitro* Ub nucleophile discharge assay data.

A Cartoon depiction of *in vitro* Ub nucleophile discharge assays. |Nu depicts the nucleophile with its electron lone pair, which in the reactions shown here is the bi-functional molecule ethanolamine.

B SDS–PAGE run under reducing conditions of samples at the beginning and end of indicated discharge reactions to show the degree of E2 auto-ubiquitylation ("-Ub") during each reaction.

C, D Quantification of Ub discharge assays with U7BR/Ubc7 and indicated Doa10 (C) and Hrd1 (D) variants. Plots of Ubc7~Ub discharge as a function of time (dots) and first-order reaction models fitted to the discharge data (lines) are shown. Values for each time point are reported as means ± standard deviation (n = 3). Insets show absolute reaction rates derived from these fits. For ease of comparison, the y-axis of these insets is the same in C–F. The "no E3" control is identical in both C and D.

E, F Quantification of Ub discharge assays with Ubc6 and indicated Doa10 (E) and Hrd1 (F) variants. Plots of Ubc6~Ub discharge as a function of time (dots) and first-order reaction models fitted to the discharge data (lines) are shown. Values for each time point are reported as means ± standard deviation (n = 3). Insets show absolute reaction rates derived from these fits. For ease of comparison, the y-axis of these insets is the same in C–F. The "no E3" control is identical in both E and F.

G Stimulation of U7BR/Ubc7 and Ubc6 discharge activities by indicated RING variants. Direct comparison of all tested E2 stimulations, shown as the ratio of rates derived from RING-catalyzed reactions and respective "no E3" controls from C and E. Values are reported as means ± standard deviation (n = 3). Significances for pairwise comparisons were determined by one-way ANOVA test; *$P < 0.05$. For clarity, only significances related to the respective "no E3" control of a given E2 are shown. Gray background indicates data shown in main figures (Figs 2E, 3A, and 4C).

Source data are available online for this figure.

© 2020 The Authors

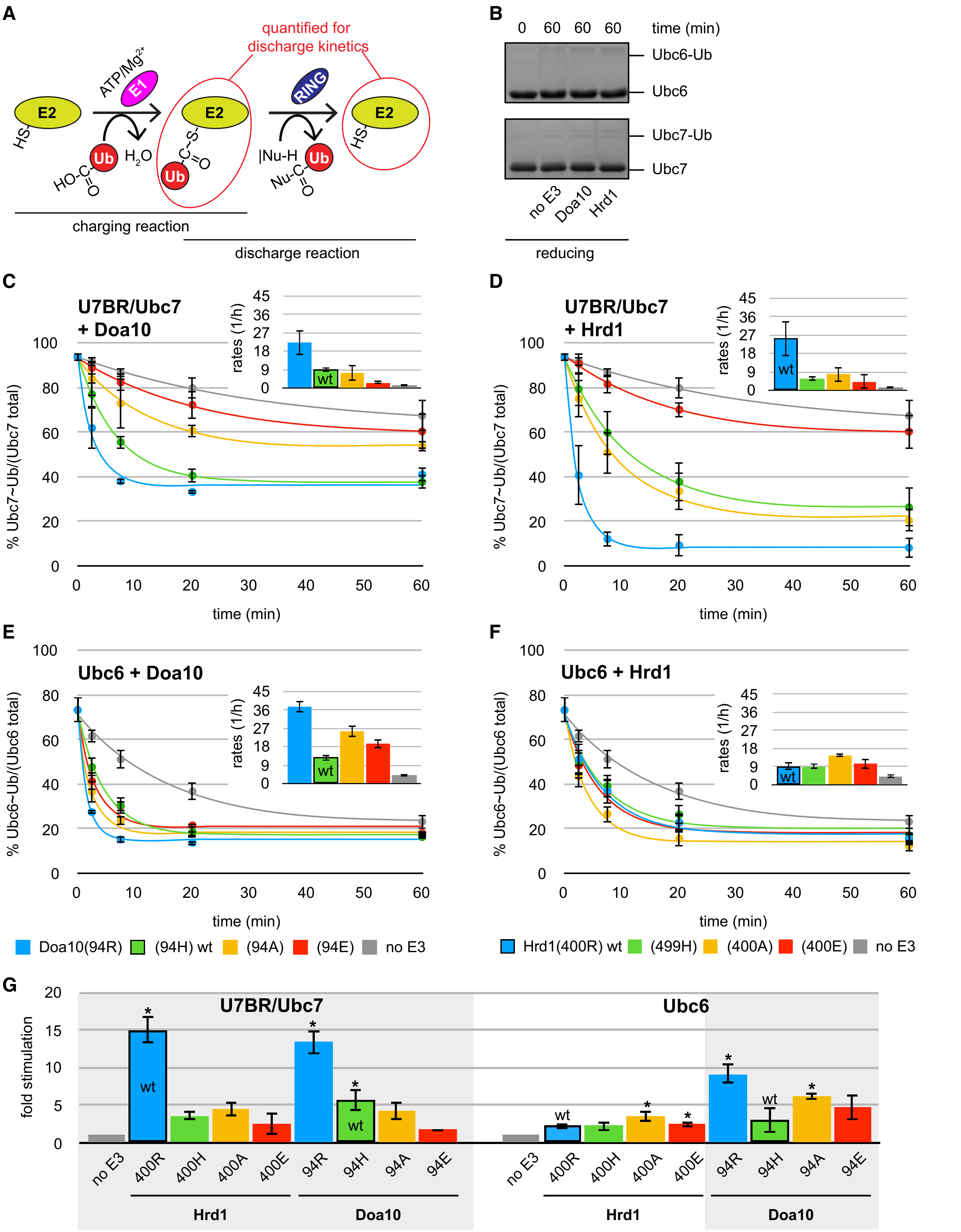

Figure EV2.

 © 2020 The Authors

Figure EV3. *In vitro* Ub chain formation assay data.

A Cartoon depiction of *in vitro* Ub chain formation assays detailing the three elongation steps monitored in this study.

B, C *In vitro* Ub chain formation assay by Ubc7 with indicated Hrd1 (B) and Doa10 (C) variants in the presence of indicated Cue1 variants. Rates for reactions of mono-Ub to di-Ub, di-Ub to tri-Ub, and tri-Ub to tetra-Ub with fluorescently labeled Ub are shown on a logarithmic scale. Values are reported as means ± standard deviation (n = 3). Significances for pairwise comparisons were determined by one-way ANOVA test; *P < 0.05. For clarity, only significances related to the respective "no E3" control of a given reaction set are shown. Gray background indicates data in part shown in main figures (Fig 3B); for easier comparison, all reactions tested are shown here side-by-side.

Source data are available online for this figure.

© 2020 The Authors

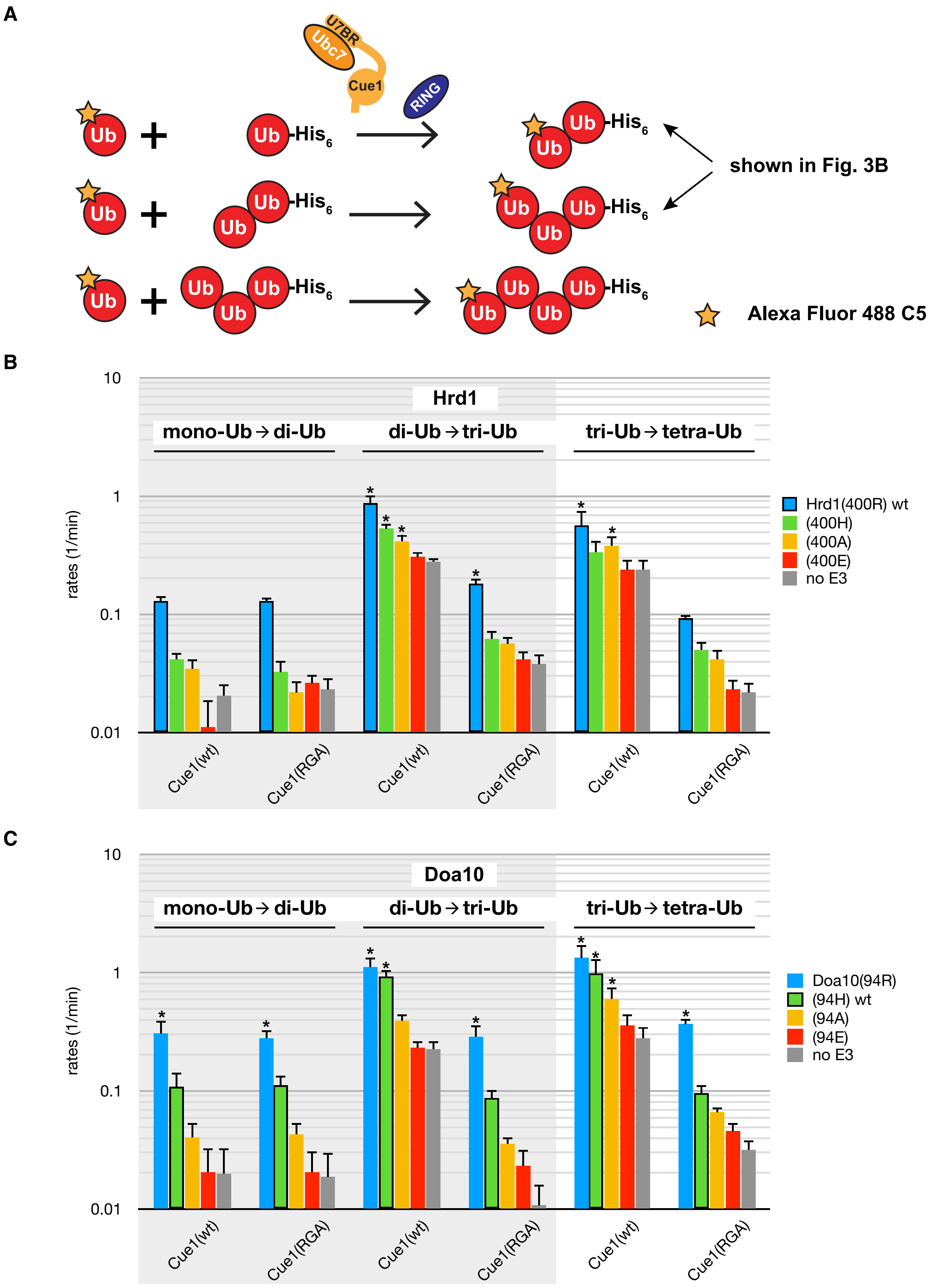

Figure EV3.

 © 2020 The Authors

Figure EV4. Protein degradation assays for the Hrd1 model substrate Hmg2-6xMyc.

A–D Protein degradation in indicated yeast strains monitored by pulse-chase experiments for the Hrd1 model substrate Hmg2-6xMyc. Values for each time point are reported as means ± standard deviation (n = 4 for wild-type strains and n = 3 for deletion strains).

E, F Data shown in A and B or in C and D, respectively, are shown in rearranged organization for clarity.

Source data are available online for this figure.

© 2020 The Authors

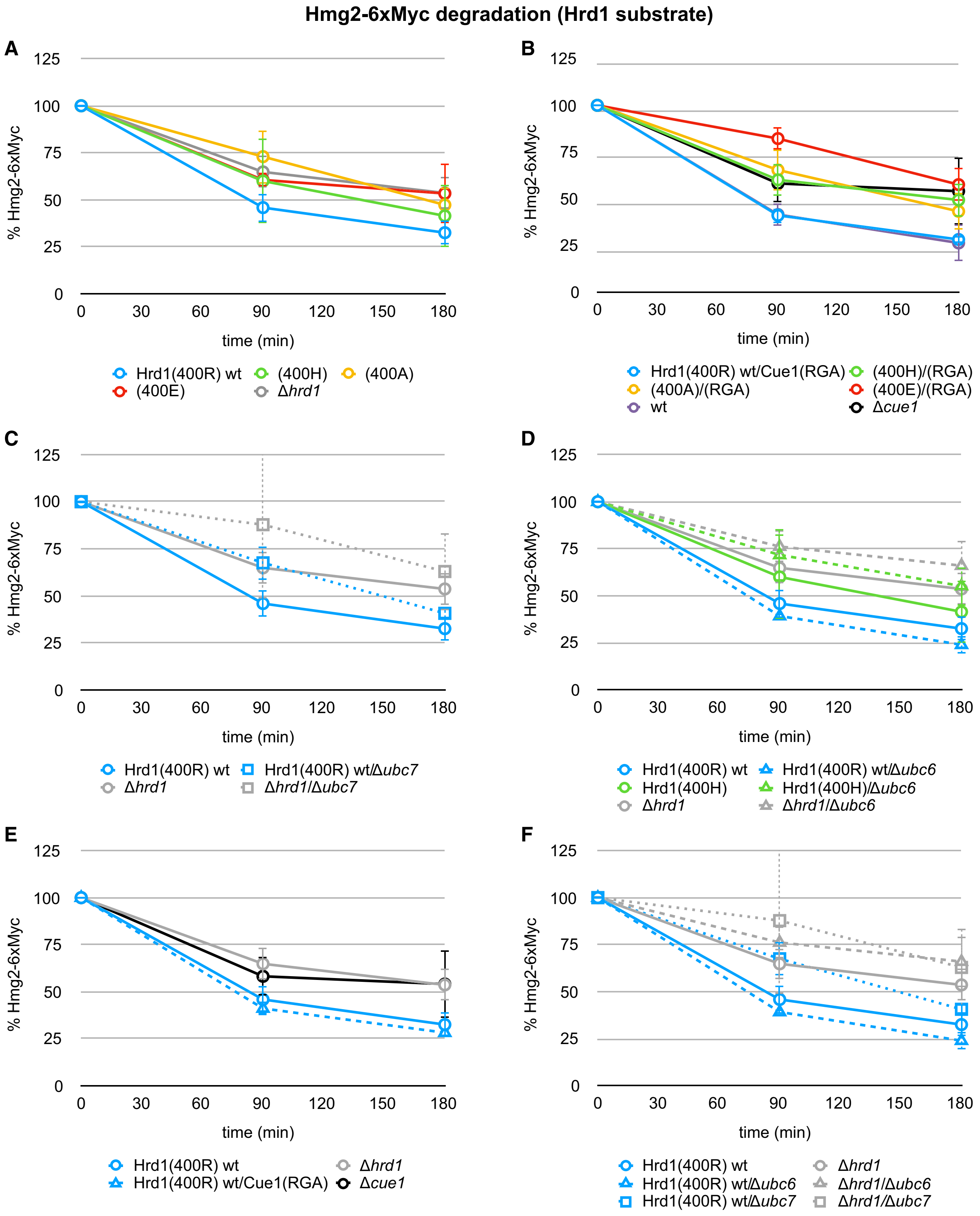

Figure EV4.

© 2020 The Authors

Figure EV5. Protein degradation assays for the Doa10 model substrate FLAG-Sbh2.

A–D Protein degradation in indicated yeast strains monitored by CHX decay assays for the Doa10 model substrate FLAG-Sbh2. Values for each time point are reported as means ± standard deviation (*n* = 3). Gray background indicates data shown in main figures (Fig 6C and F); for better comparison, all assays are shown here side-by-side.

E, F Data shown in A and B or in C and D, respectively, are shown in rearranged organization for clarity.

Source data are available online for this figure.

© 2020 The Authors

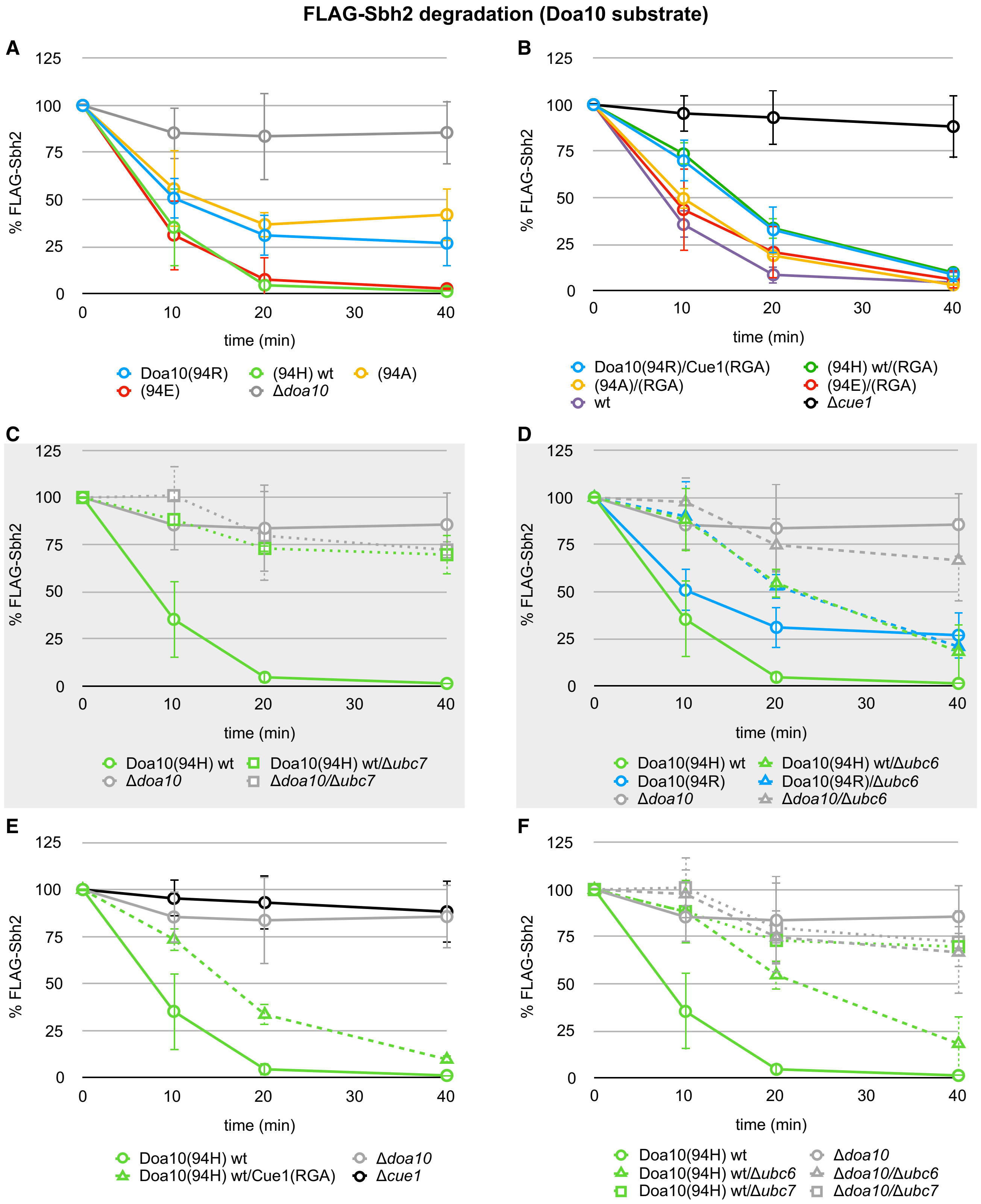

Figure EV5.

© 2020 The Authors
