# Supplementary material for: Who with whom: functional coordination of E2 enzymes by RING E3 ligases during poly‐ubiquitylation
Source: EMBO J. 2020 Oct 5;39(22):e104863. doi: 10.15252/embj.2020104863 (PMC7667886; doi:10.15252/embj.2020104863)
Supplement: Supplementary file 3 — Source Data for Expanded View and Appendix [file EMBJ-39-e104863-s008.zip › 2020-104863_SourceData/2020-104863_SourceData_ExpandedView/2020-104863_SourceDataForFigEV5.pdf]

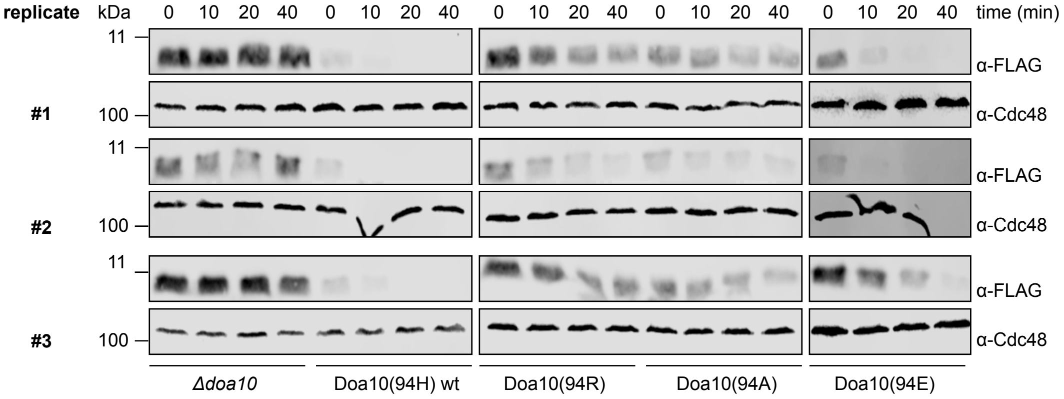

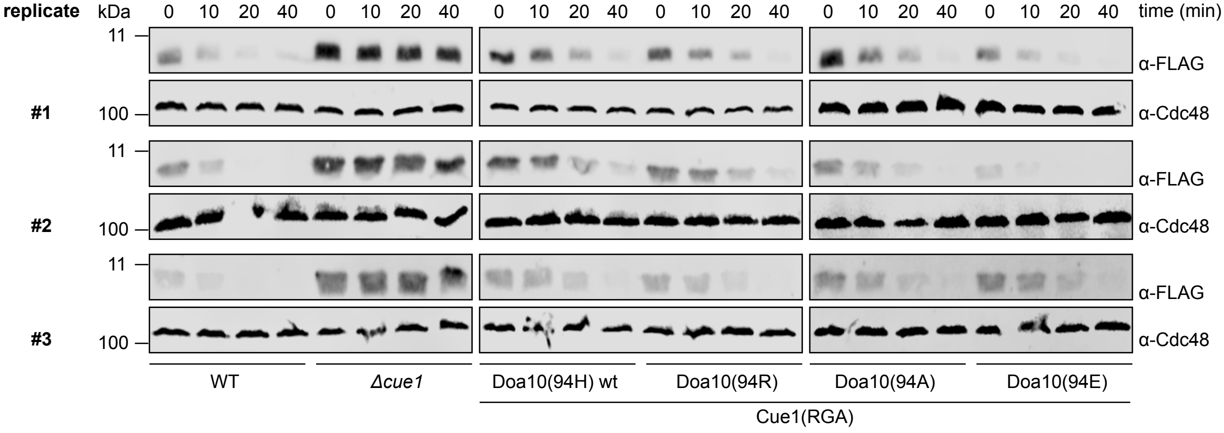

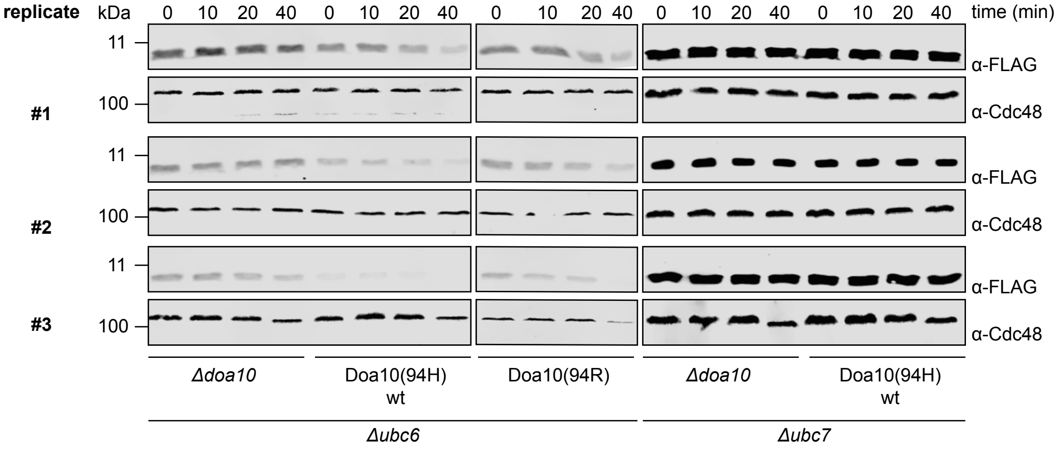

Source Data for Fig. EV5

Protein degradation in indicated yeast strains monitored by CHX decay assays for the Doa10 model substrate FLAG-Sbh2. Immunoblots are shown (n = 3), which are the basis for quantifications reported in Fig. EV5. Replicates for the *Δdoa10*, Doa10(94H) wt and Doa10(94R) strains as well as all *Δubc6* and *Δdubc7* strains are identical to the ones shown in Source Data for Fig. 6 panel C.
