# Supplementary material for: Who with whom: functional coordination of E2 enzymes by RING E3 ligases during poly‐ubiquitylation
Source: EMBO J. 2020 Oct 5;39(22):e104863. doi: 10.15252/embj.2020104863 (PMC7667886; doi:10.15252/embj.2020104863)
Supplement: Supplementary file 3 — Source Data for Expanded View and Appendix [file EMBJ-39-e104863-s008.zip › 2020-104863_SourceData/2020-104863_SourceData_ExpandedView/2020-104863_SourceDataForFigEV4.pdf]

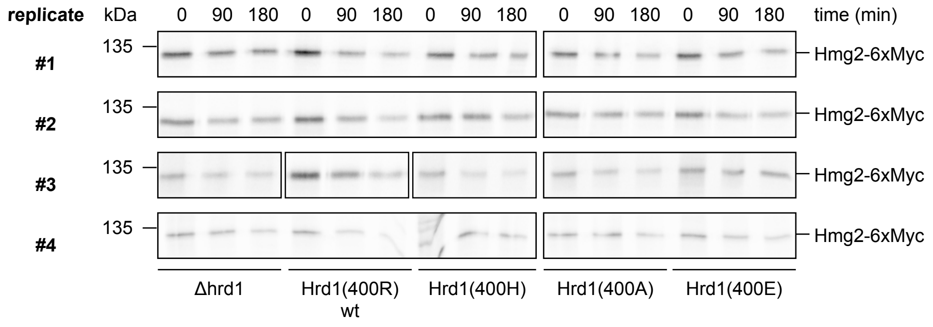

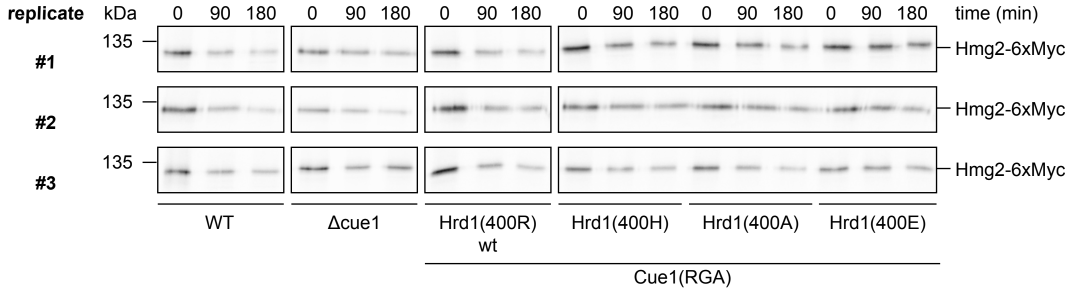

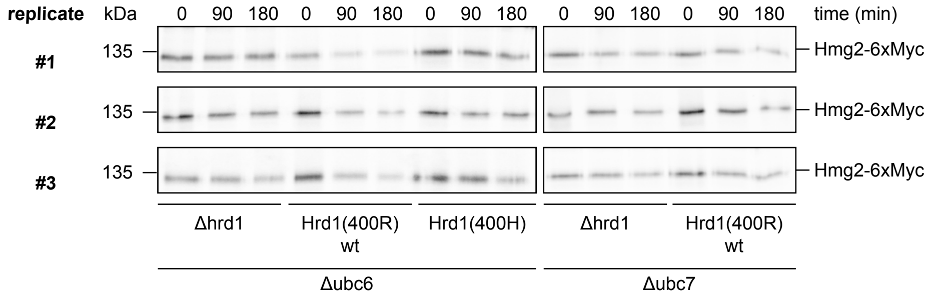

Source Data for Fig. EV4
Protein degradation in indicated yeast strains monitored by pulse-chase experiments for the Hrd1 model substrate Hmg2-6xMyc. Immunoblots are shown (n = 4 for wild-type strains and n = 3 for deletion strains), which are the basis for quantifications reported in Fig. EV4.
