# Supplementary material for: Who with whom: functional coordination of E2 enzymes by RING E3 ligases during poly‐ubiquitylation
Source: EMBO J. 2020 Oct 5;39(22):e104863. doi: 10.15252/embj.2020104863 (PMC7667886; doi:10.15252/embj.2020104863)
Supplement: Supplementary file 3 — Source Data for Expanded View and Appendix [file EMBJ-39-e104863-s008.zip › 2020-104863_SourceData/2020-104863_SourceData_ExpandedView/2020-104863_SourceDataForFigEV3.pdf]

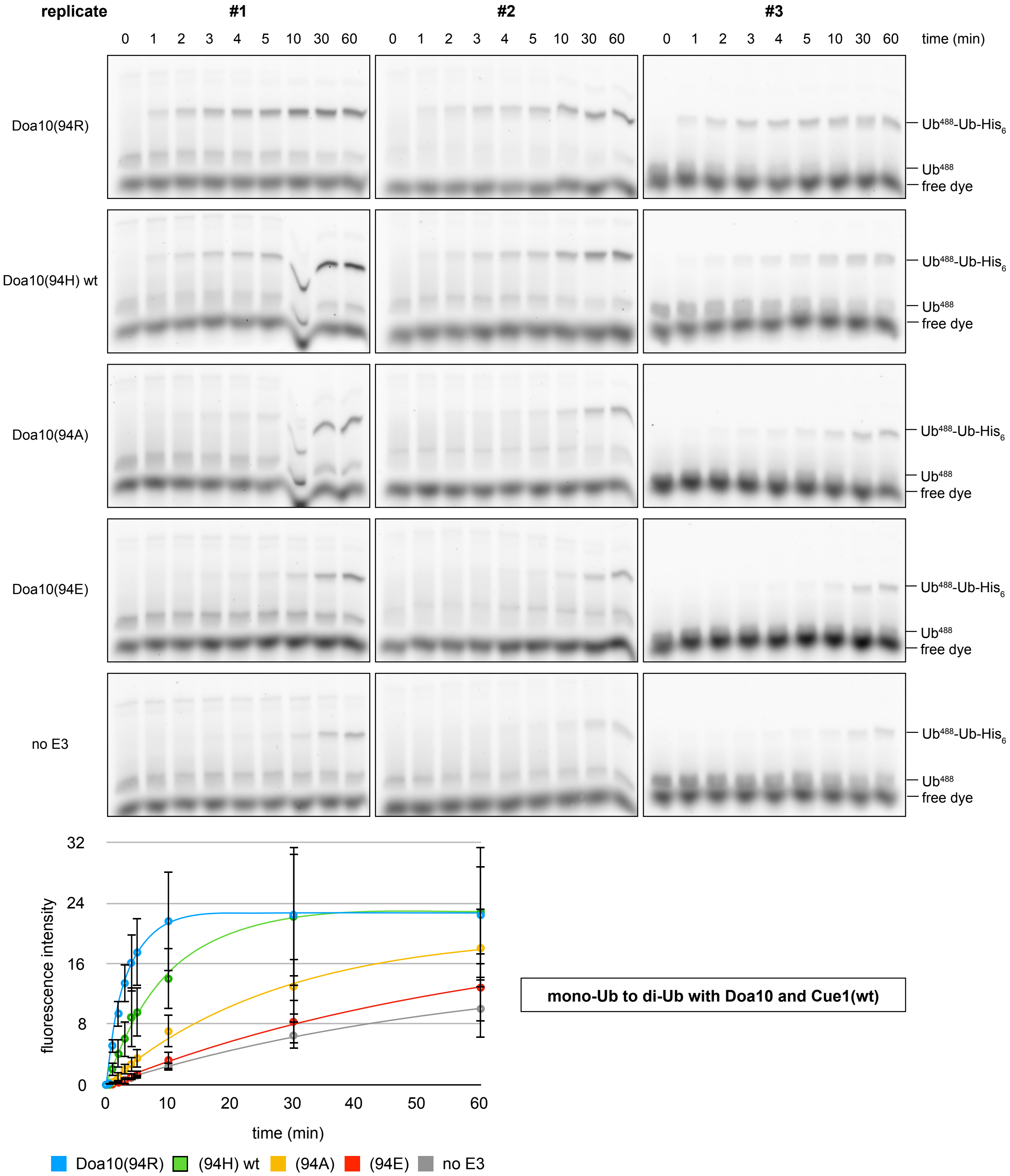

Source Data for Fig. EV3 - Chain formation assay data for mono-Ub to di-Ub reaction with Doa10 variants and Cue1(wt). Top: Fluorescent scans for triplicate experiments of mono-Ub to di-Ub reaction with Ubc7, Cue1(wt) with indicated Doa10 variants; Ub^{488} = Alexa Fluor 488 C5-labeled Ub(S20C). Bottom: Plots of fluorescent intensity of product band as a function of time (dots) and first-order reaction models fitted to the data (lines). Values for each time point are reported as means ± standard deviation. Rates reported in Fig. 3B and EV3 are derived from the fits reported here.

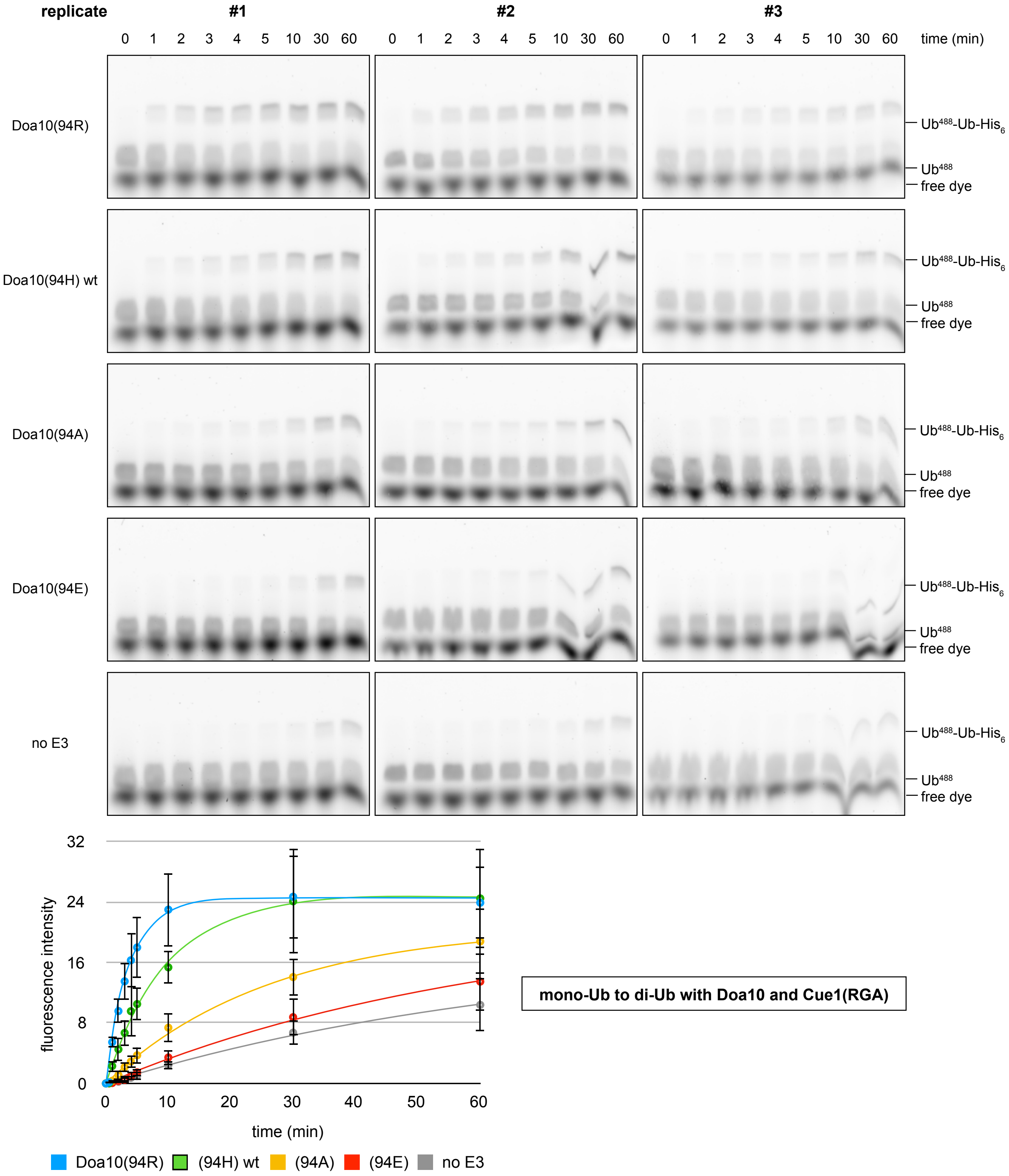

Source Data for Fig. EV3 - Chain formation assay data for mono-Ub to di-Ub reaction with Doa10 variants and Cue1(RGA).
Top: Fluorescent scans for triplicate experiments of mono-Ub to di-Ub reaction with Ubc7, Cue1(RGA) with indicated Doa10 variants; Ub^{488} = Alexa Fluor 488 C5-labeled Ub(S20C). Bottom: Plots of fluorescent intensity of product band as a function of time (dots) and first-order reaction models fitted to the data (lines). Values for each time point are reported as means ± standard deviation. Rates reported in Fig. 3B and EV3 are derived from the fits reported here.

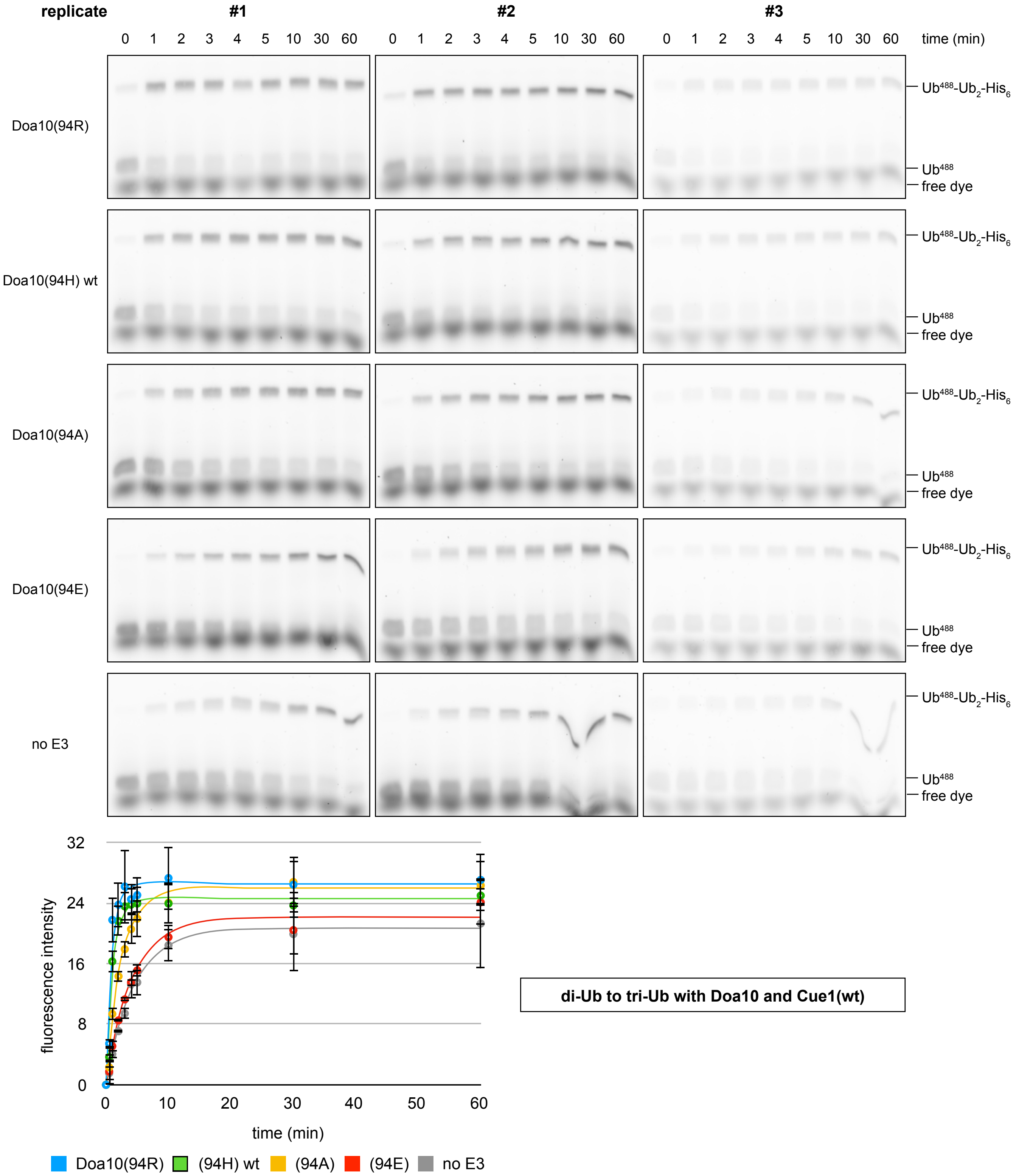

Source Data for Fig. EV3 - Chain formation assay data for di-Ub to tri-Ub reaction with Doa10 variants and Cue1(wt).
Top: Fluorescent scans for triplicate experiments of di-Ub to tri-Ub reaction with Ubc7, Cue1(wt) with indicated Doa10 variants; Ub^{488} = Alexa Fluor 488 C5-labeled Ub(S20C). Bottom: Plots of fluorescent intensity of product band as a function of time (dots) and first-order reaction models fitted to the data (lines). Values for each time point are reported as means ± standard deviation. Rates reported in Fig. 3B and EV3 are derived from the fits reported here.

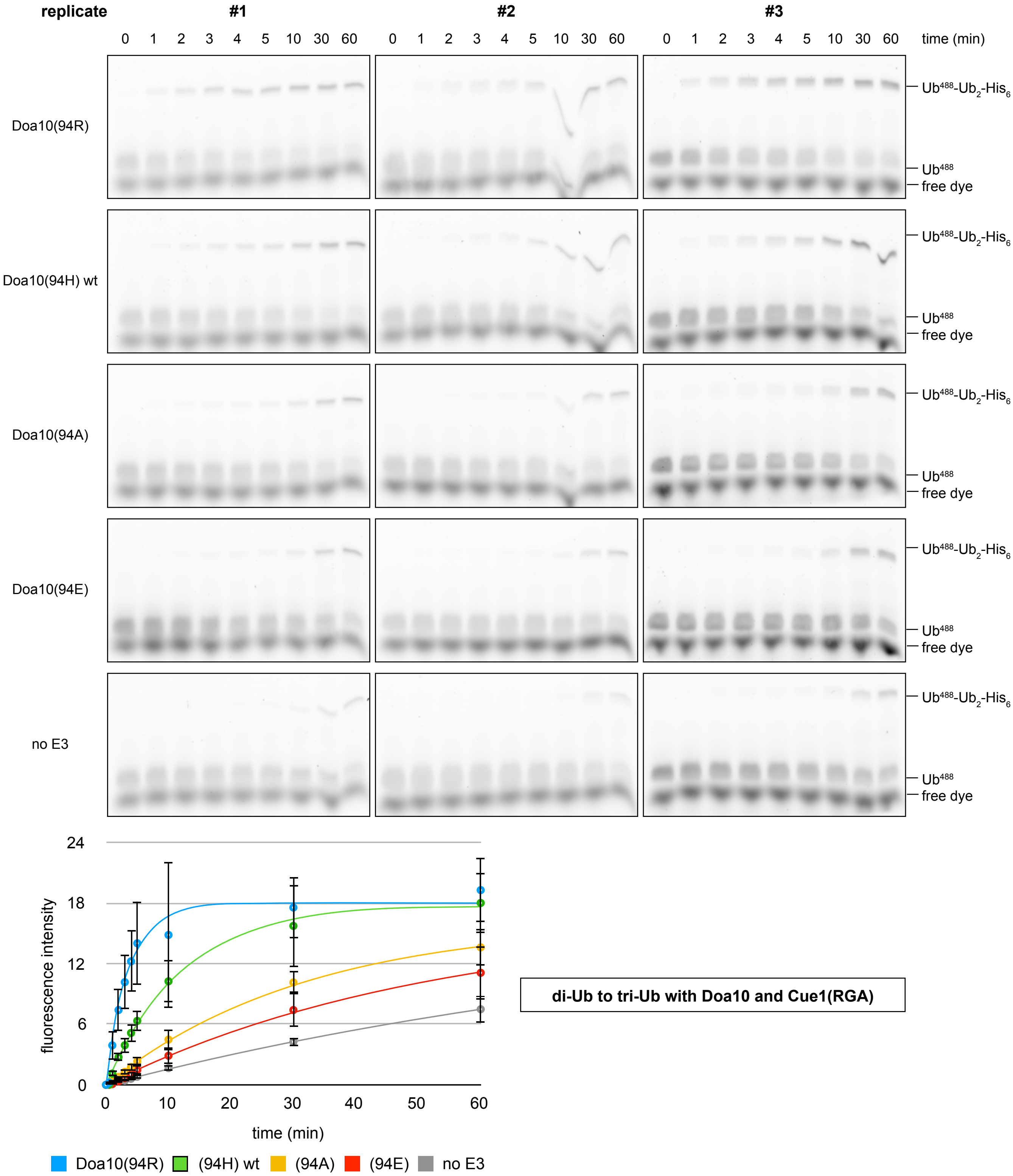

Source Data for Fig. EV3 - Chain formation assay data for di-Ub to tri-Ub reaction with Doa10 variants and Cue1(RGA).
Top: Fluorescent scans for triplicate experiments of di-Ub to tri-Ub reaction with Ubc7, Cue1(RGA) with indicated Doa10 variants; Ub^{488} = Alexa Fluor 488 C5-labeled Ub(S20C). Bottom: Plots of fluorescent intensity of product band as a function of time (dots) and first-order reaction models fitted to the data (lines). Values for each time point are reported as means ± standard deviation. Rates reported in Fig. 3B and EV3 are derived from the fits reported here.

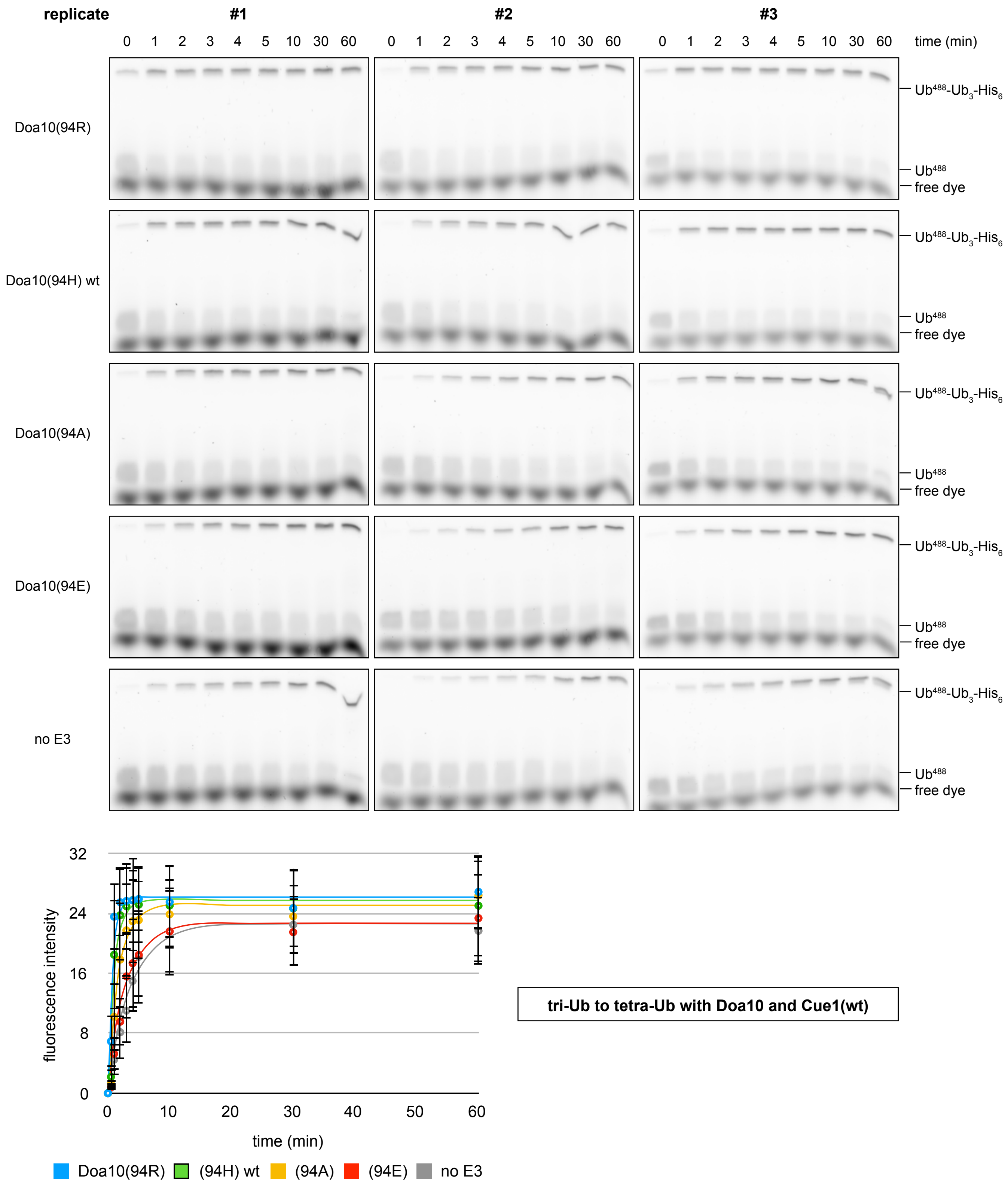

Source Data for Fig. EV3 - Chain formation assay data for tri-Ub to tetra-Ub reaction with Doa10 variants and Cue1(wt). Top: Fluorescent scans for triplicate experiments of tri-Ub to tetra-Ub reaction with Ubc7, Cue1(wt) with indicated Doa10 variants; Ub^{488} = Alexa Fluor 488 C5-labeled Ub(S20C). Bottom: Plots of fluorescent intensity of product band as a function of time (dots) and first-order reaction models fitted to the data (lines). Values for each time point are reported as means ± standard deviation. Rates reported in Fig. 3B and EV3 are derived from the fits reported here.

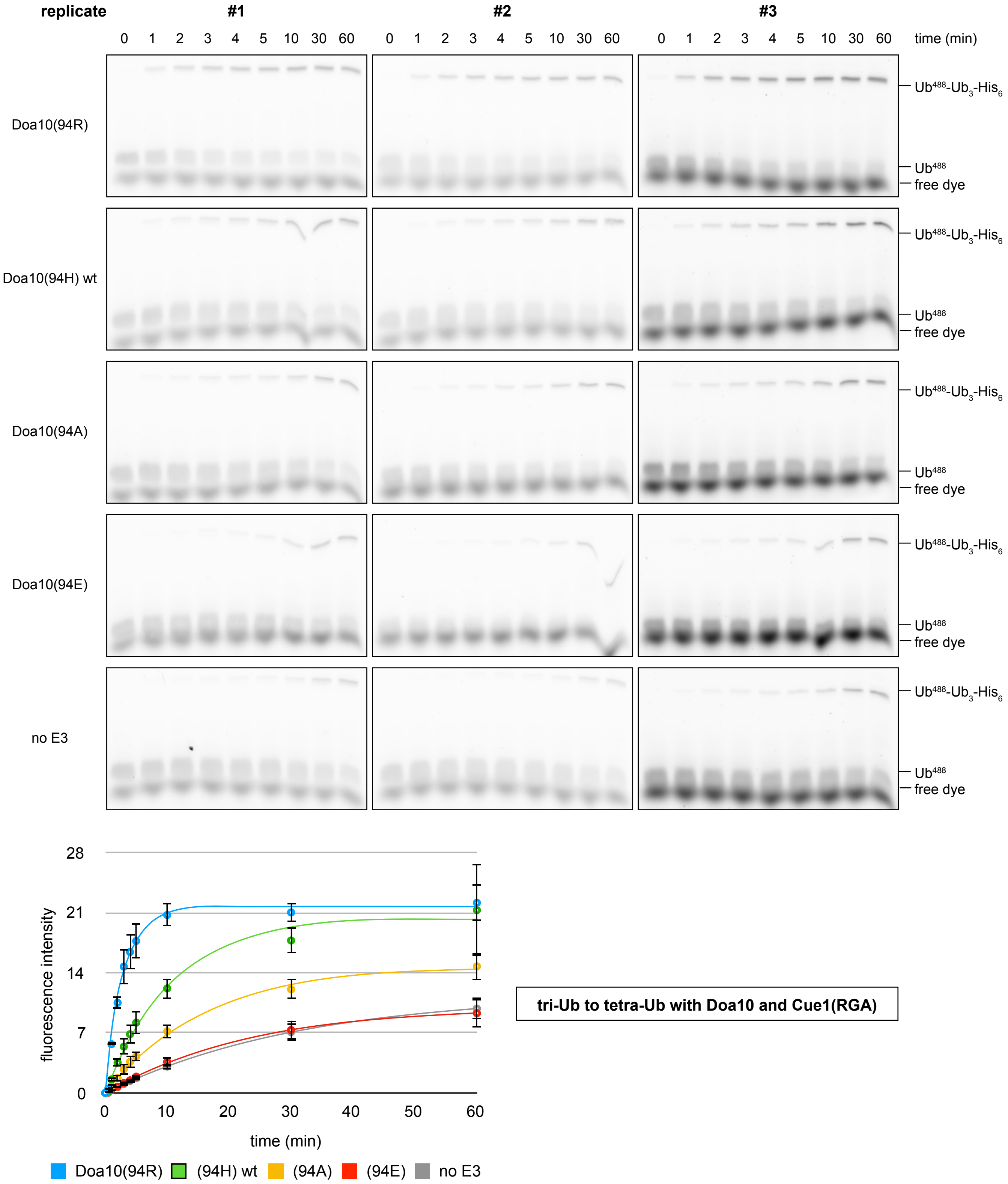

Source Data for Fig. EV3 - Chain formation assay data for tri-Ub to tetra-Ub reaction with Doa10 variants and Cue1(RGA).
Top: Fluorescent scans for triplicate experiments of tri-Ub to tetra-Ub reaction with Ubc7, Cue1(RGA) with indicated Doa10 variants; Ub^{488} = Alexa Fluor 488 C5-labeled Ub(S20C). Bottom: Plots of fluorescent intensity of product band as a function of time (dots) and first-order reaction models fitted to the data (lines). Values for each time point are reported as means ± standard deviation. Rates reported in Fig. 3B and EV3 are derived from the fits reported here.

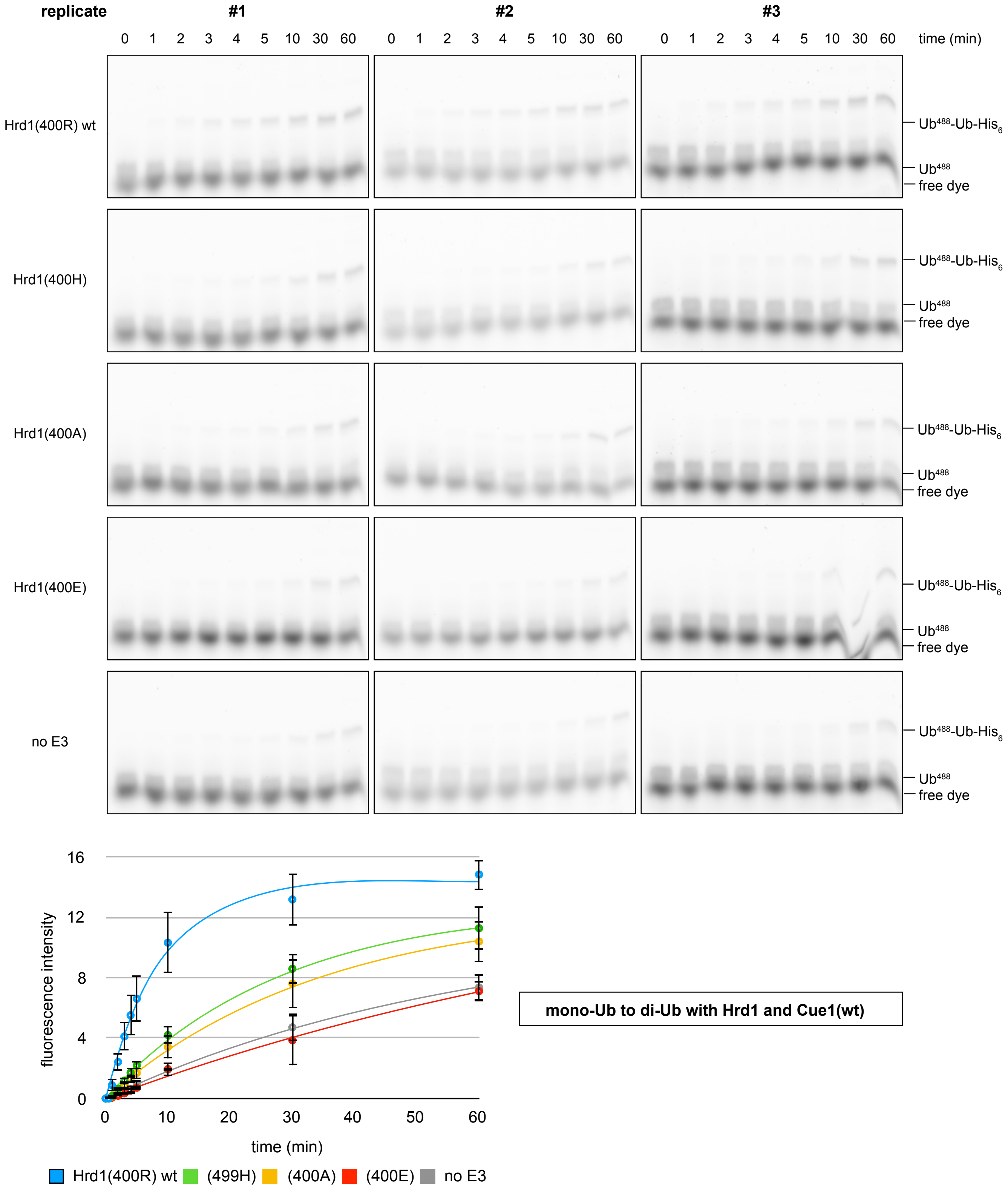

Source Data for Fig. EV3 - Chain formation assay data for mono-Ub to di-Ub reaction with Hrd1 variants and Cue1(wt). Top: Fluorescent scans for triplicate experiments of mono-Ub to di-Ub reaction with Ubc7, Cue1(wt) with indicated Hrd1 variants; Ub^{488} = Alexa Fluor 488 C5-labeled Ub(S20C). Bottom: Plots of fluorescent intensity of product band as a function of time (dots) and first-order reaction models fitted to the data (lines). Values for each time point are reported as means ± standard deviation. Rates reported in Fig. 3B and EV3 are derived from the fits reported here.

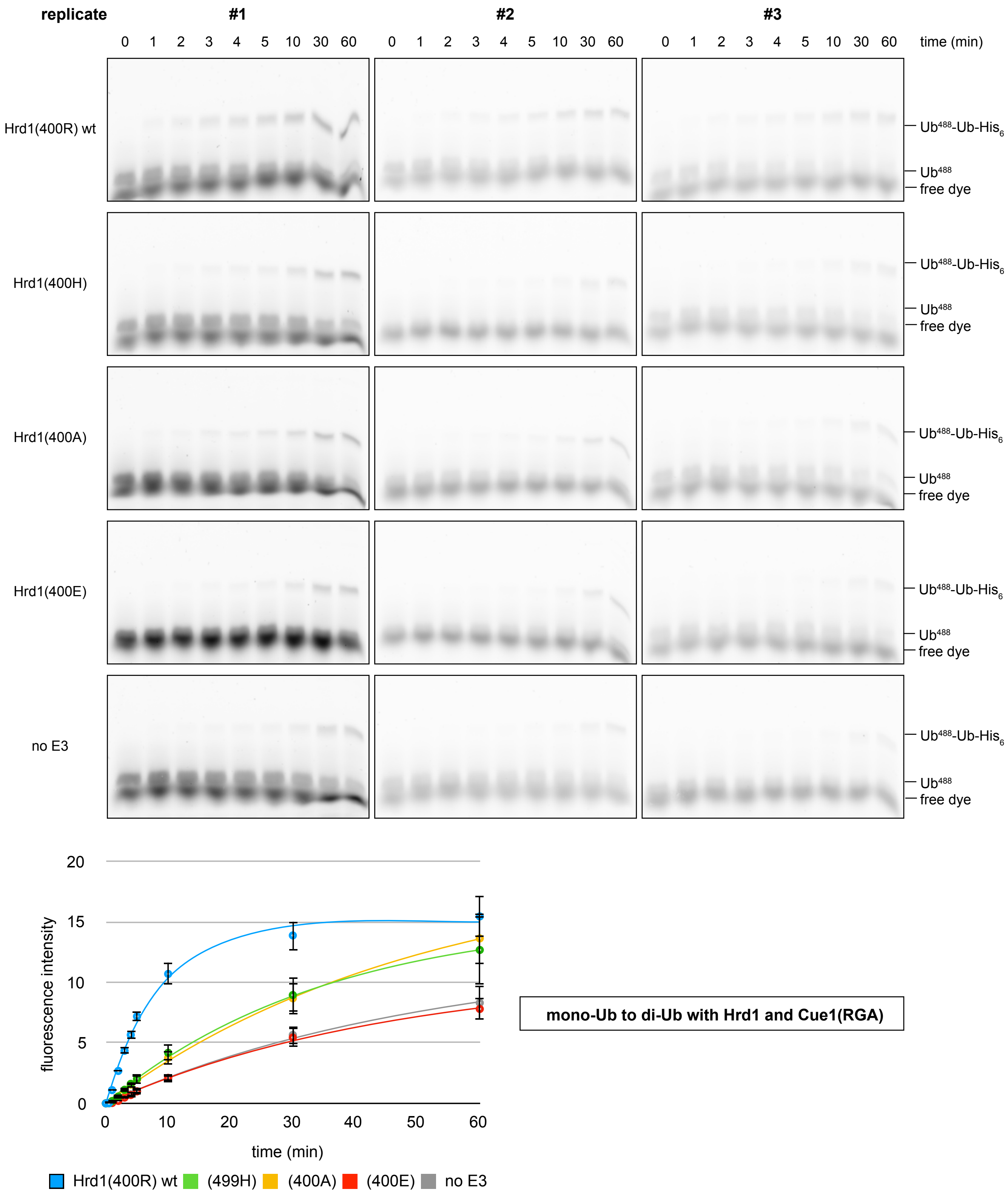

Source Data for Fig. EV3 - Chain formation assay data for mono-Ub to di-Ub reaction with Hrd1 variants and Cue1(RGA).
Top: Fluorescent scans for triplicate experiments of mono-Ub to di-Ub reaction with Ubc7, Cue1(wt) with indicated Hrd1 variants; Ub^{488} = Alexa Fluor 488 C5-labeled Ub(S20C). Bottom: Plots of fluorescent intensity of product band as a function of time (dots) and first-order reaction models fitted to the data (lines). Values for each time point are reported as means ± standard deviation. Rates reported in Fig. 3B and EV3 are derived from the fits reported here.

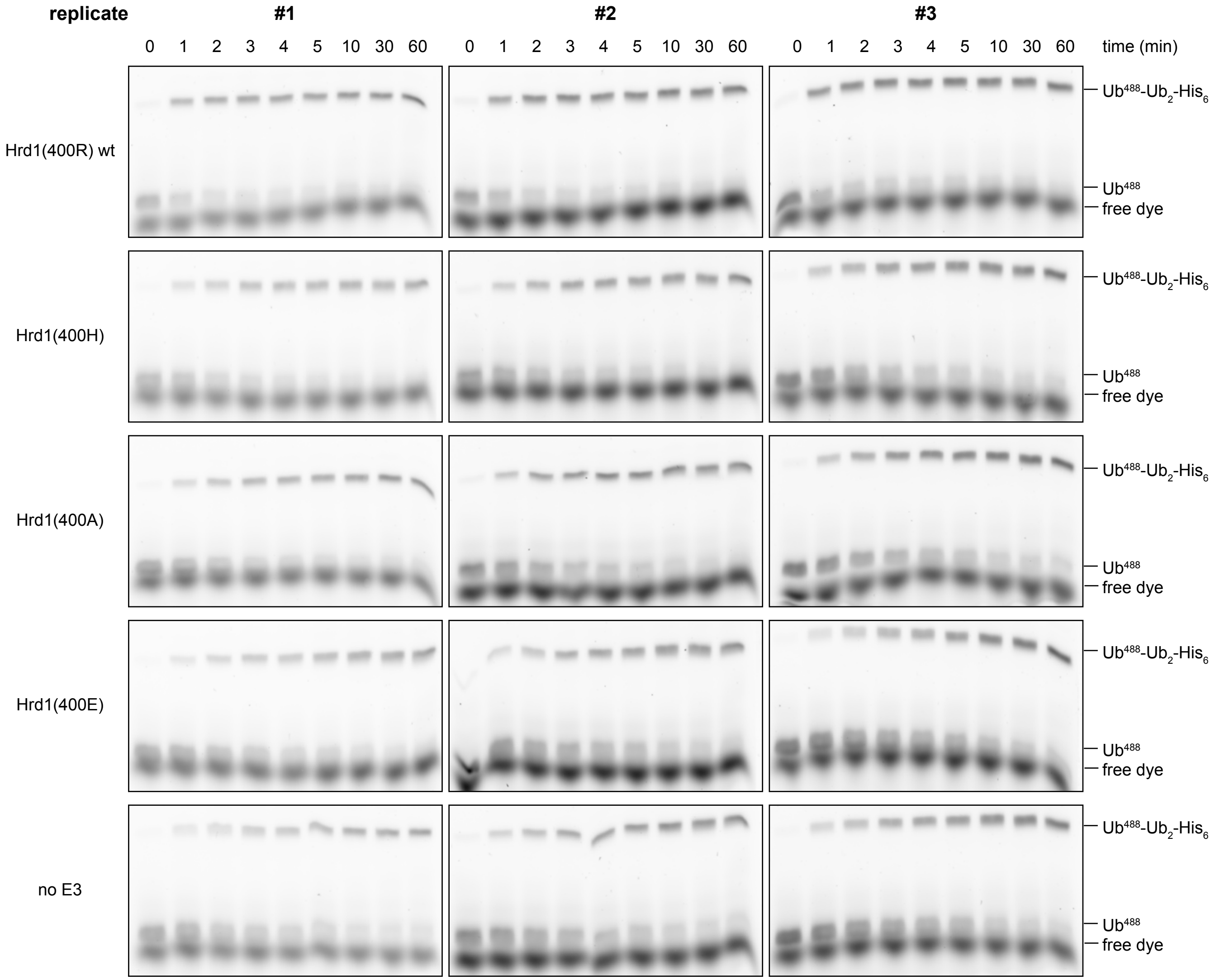

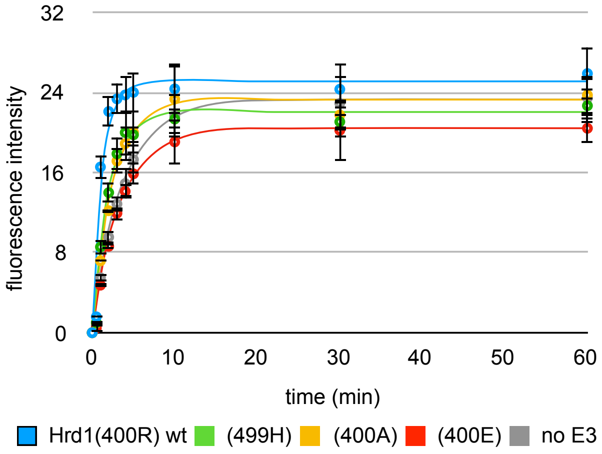

di-Ub to tri-Ub with Hrd1 and Cue1(wt)

Source Data for Fig. EV3 - Chain formation assay data for di-Ub to tri-Ub reaction with Hrd1 variants and Cue1(wt).
Top: Fluorescent scans for triplicate experiments of mono-Ub to di-Ub reaction with Ubc7, Cue1(wt) with indicated Hrd1 variants; Ub^{488} = Alexa Fluor 488 C5-labeled Ub(S20C). Bottom: Plots of fluorescent intensity of product band as a function of time (dots) and first-order reaction models fitted to the data (lines). Values for each time point are reported as means ± standard deviation. Rates reported in Fig. 3B and EV3 are derived from the fits reported here.

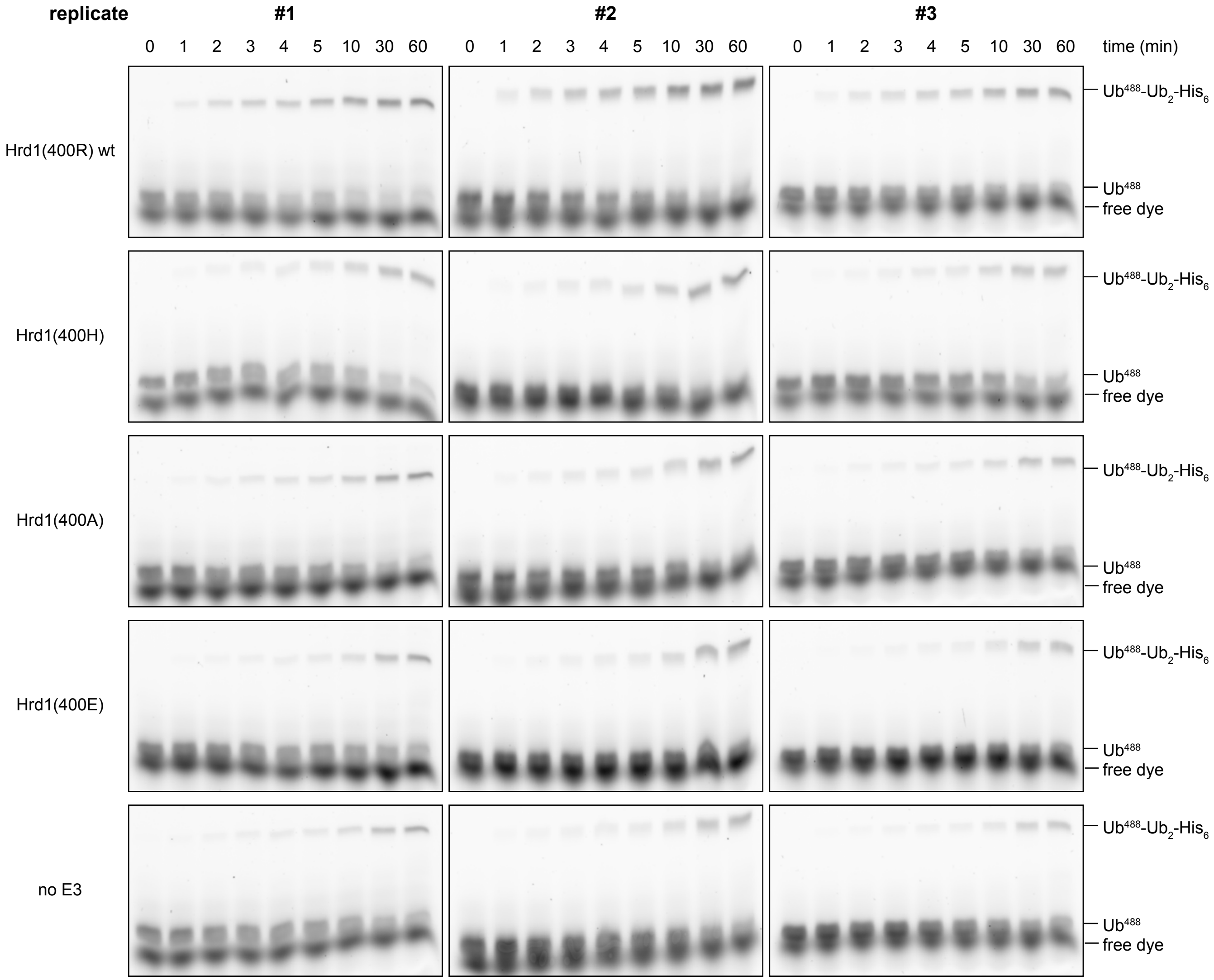

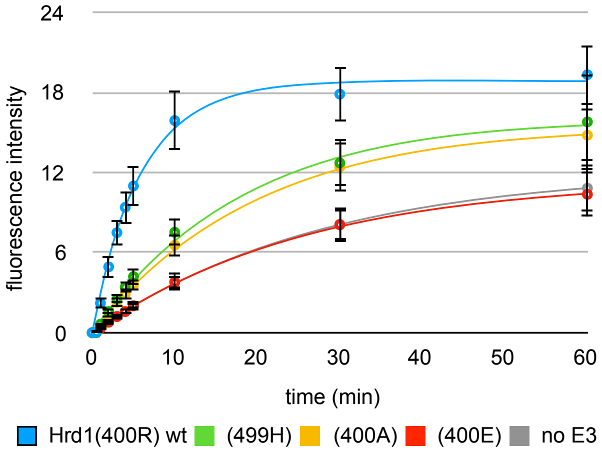

di-Ub to tri-Ub with Hrd1 and Cue1(RGA)

Source Data for Fig. EV3 - Chain formation assay data for di-Ub to tri-Ub reaction with Hrd1 variants and Cue1(RGA).
Top: Fluorescent scans for triplicate experiments of di-Ub to tri-Ub reaction with Ubc7, Cue1(RGA) with indicated Hrd1 variants; Ub^{488} = Alexa Fluor 488 C5-labeled Ub(S20C). Bottom: Plots of fluorescent intensity of product band as a function of time (dots) and first-order reaction models fitted to the data (lines). Values for each time point are reported as means ± standard deviation. Rates reported in Fig. 3B and EV3 are derived from the fits reported here.

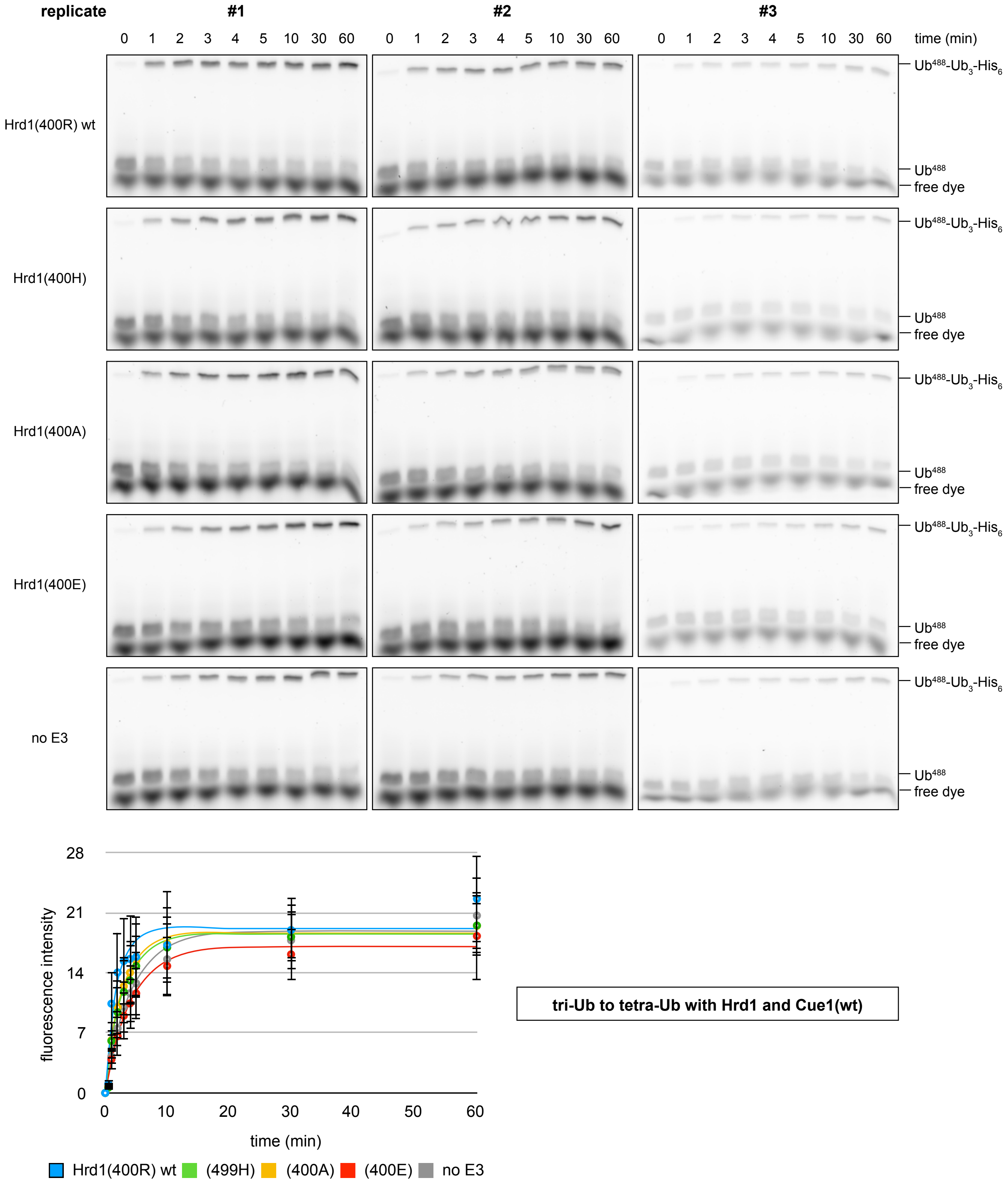

Source Data for Fig. EV3 - Chain formation assay data for tri-Ub to tetra-Ub reaction with Hrd1 variants and Cue1(wt). Top: Fluorescent scans for triplicate experiments of tri-Ub to tetra-Ub reaction with Ubc7, Cue1(wt) with indicated Hrd1 variants; Ub^{488} = Alexa Fluor 488 C5-labeled Ub(S20C). Bottom: Plots of fluorescent intensity of product band as a function of time (dots) and first-order reaction models fitted to the data (lines). Values for each time point are reported as means ± standard deviation. Rates reported in Fig. 3B and EV3 are derived from the fits reported here.

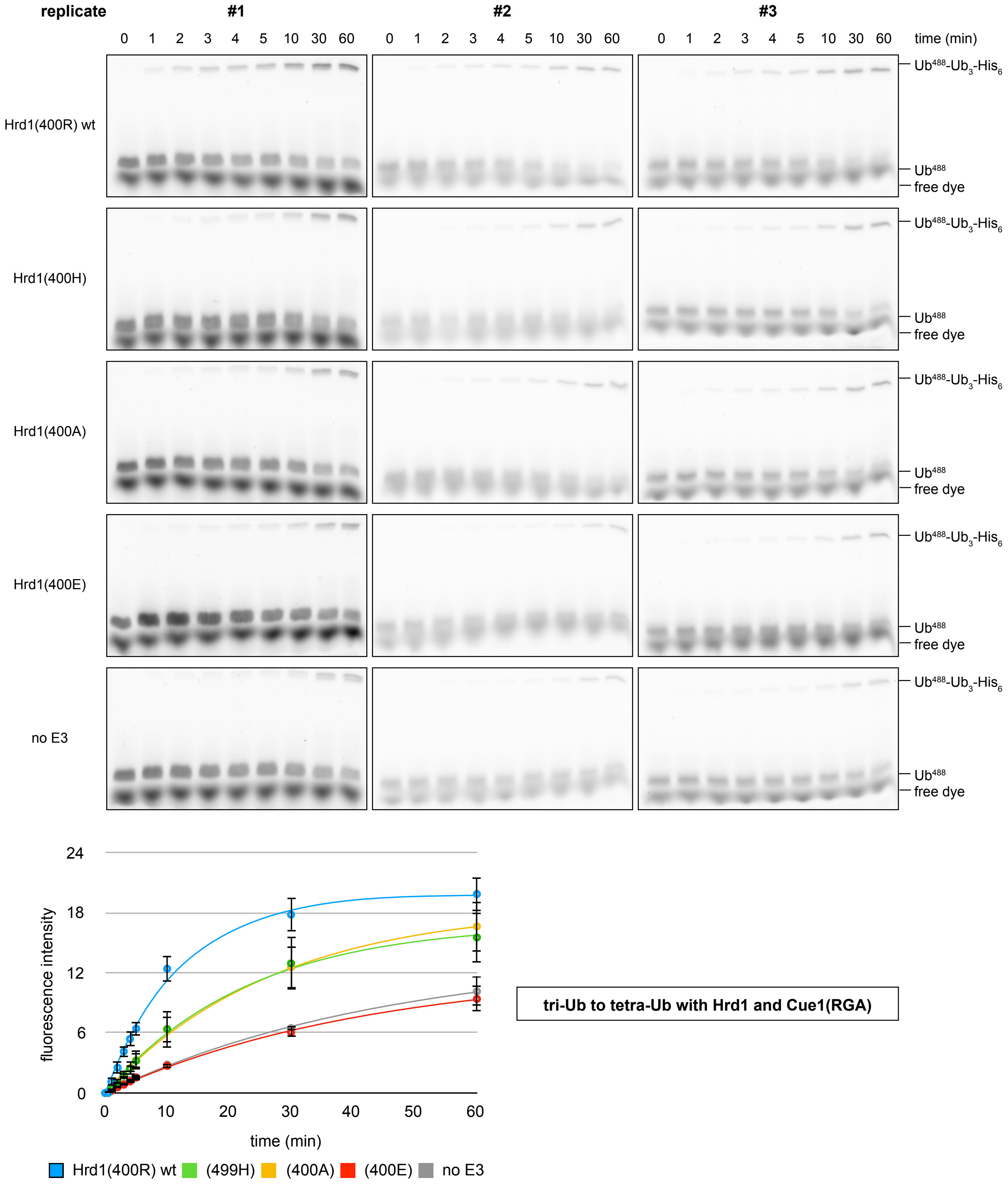

Source Data for Fig. EV3 - Chain formation assay data for tri-Ub to tetra-Ub reaction with Hrd1 variants and Cue1(RGA). Top: Fluorescent scans for triplicate experiments of tri-Ub to tetra-Ub reaction with Ubc7, Cue1(RGA) with indicated Hrd1 variants; Ub^{488} = Alexa Fluor 488 C5-labeled Ub(S20C). Bottom: Plots of fluorescent intensity of product band as a function of time (dots) and first-order reaction models fitted to the data (lines). Values for each time point are reported as means ± standard deviation. Rates reported in Fig. 3B and EV3 are derived from the fits reported here.
