# Supplementary material for: Who with whom: functional coordination of E2 enzymes by RING E3 ligases during poly‐ubiquitylation
Source: EMBO J. 2020 Oct 5;39(22):e104863. doi: 10.15252/embj.2020104863 (PMC7667886; doi:10.15252/embj.2020104863)
Supplement: Supplementary file 3 — Source Data for Expanded View and Appendix [file EMBJ-39-e104863-s008.zip › 2020-104863_SourceData/2020-104863_SourceData_ExpandedView/2020-104863_SourceDataForFigEV2.pdf]

A

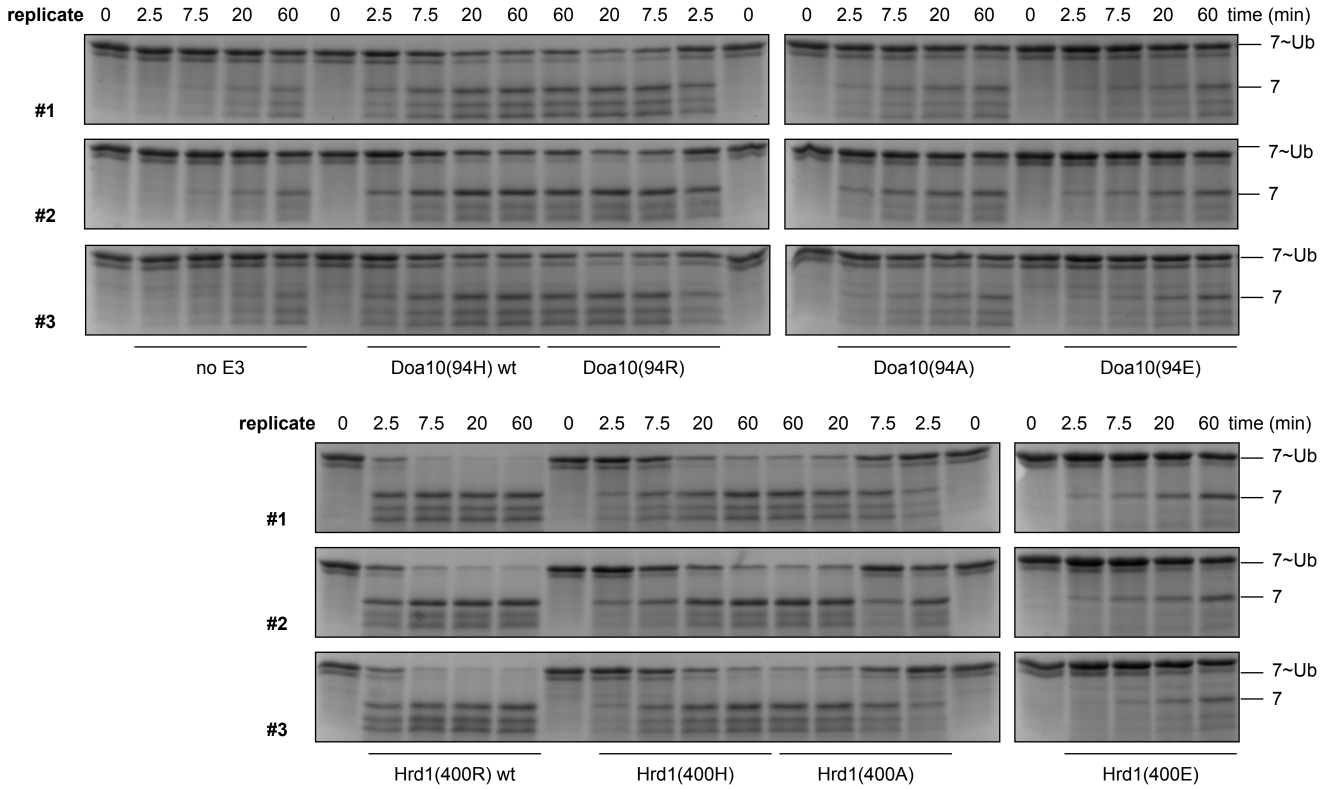

B

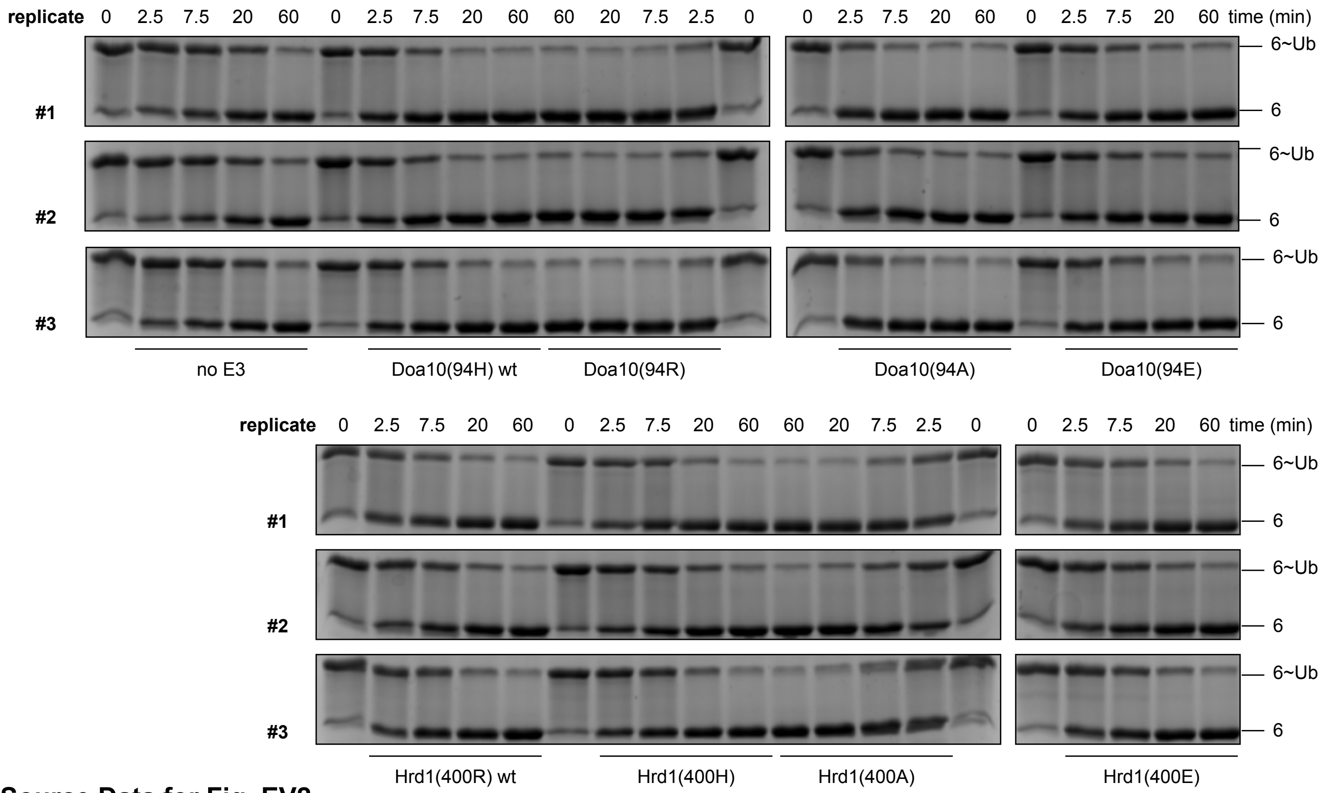

Source Data for Fig. EV2

Source Data for Fig. EV2

A + B *In vitro* Ub nucleophile discharge assays with U7BR/Ubc7 (A) and Ubc6 (B). Coomassie-stained SDS-PAGE gels under non-reducing conditions are shown (n = 3). Reactions contain ethanolamine as nucleophile and indicated RING variants. “0” time points were taken from the same sample; 6 = Ubc6, 7= Ubc7. Gels shown here are the basis for quantifications and derived rates reported in Fig. EV2 and fold stimulation reported in Fig. 3A, 4C and EV2G. Replicates for the “no E3” controls and the samples with Hrd1(400R) wt are identical to the ones shown in Source Data for Fig. 2 panel F.
