# Supplementary material for: Who with whom: functional coordination of E2 enzymes by RING E3 ligases during poly‐ubiquitylation
Source: EMBO J. 2020 Oct 5;39(22):e104863. doi: 10.15252/embj.2020104863 (PMC7667886; doi:10.15252/embj.2020104863)
Supplement: Supplementary file 3 — Source Data for Expanded View and Appendix [file EMBJ-39-e104863-s008.zip › 2020-104863_SourceData/2020-104863_SourceData_Appendix/2020-104863_SourceDataForAppendixFigS3.pdf]

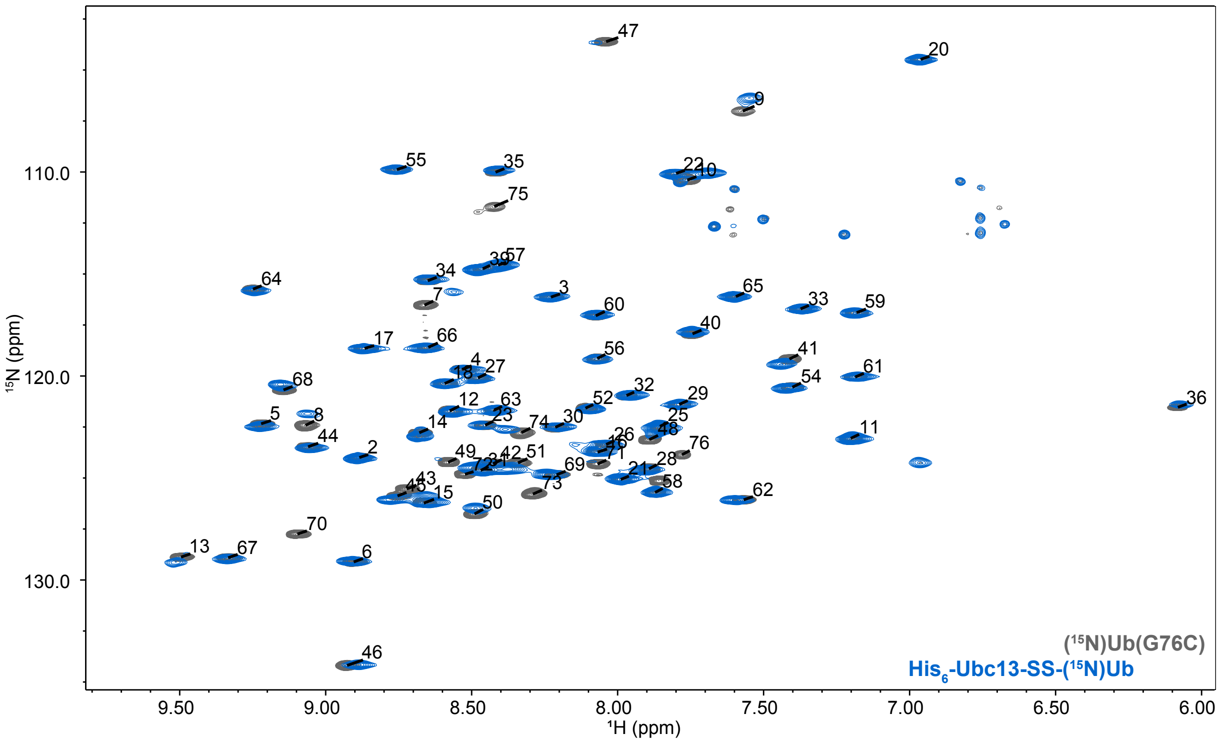

Source Data for Appendix Fig. S3

Overlaid HSQC-TROSY spectra of free (^{15}N)Ub(G76C) (775 µM - grey spectrum) and of (^{15}N)Ub(G76C) conjugated via disulfide bond to His$_6$-Ubc13 (775 µM - blue spectrum). Residue assignments for free Ub(G76C) are shown. For clarity, different contour levels for the spectra of the free and the conjugated Ub are displayed. Spectra are the basis for CSPs reported in Appendix Fig. S3.
