# Supplementary material for: Who with whom: functional coordination of E2 enzymes by RING E3 ligases during poly‐ubiquitylation
Source: EMBO J. 2020 Oct 5;39(22):e104863. doi: 10.15252/embj.2020104863 (PMC7667886; doi:10.15252/embj.2020104863)
Supplement: Supplementary file 3 — Source Data for Expanded View and Appendix [file EMBJ-39-e104863-s008.zip › 2020-104863_SourceData/2020-104863_SourceData_Appendix/2020-104863_SourceDataForAppendixFigS1.pdf]

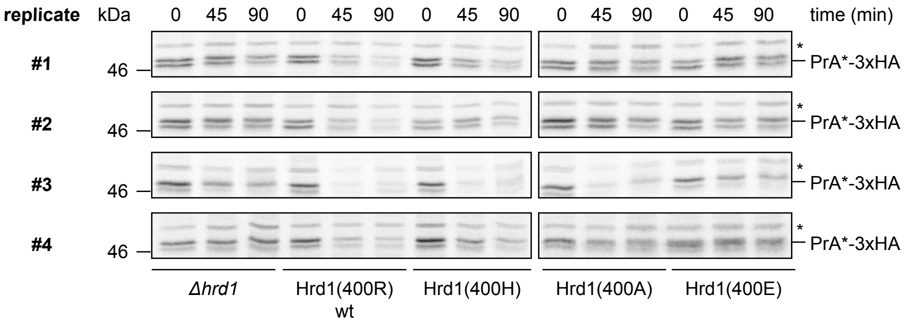

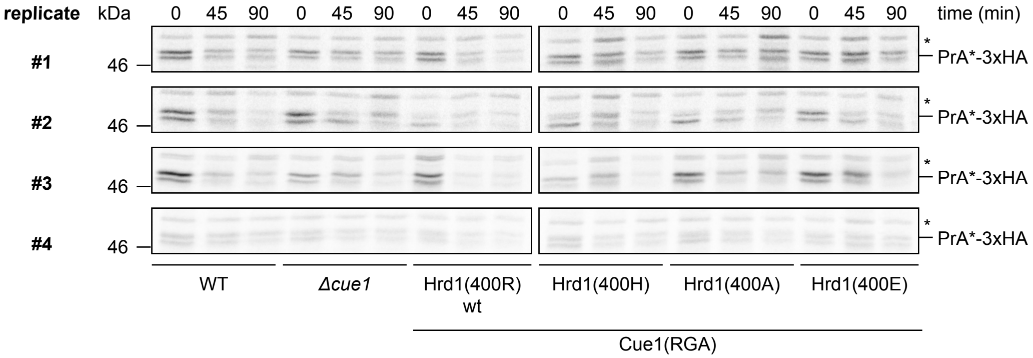

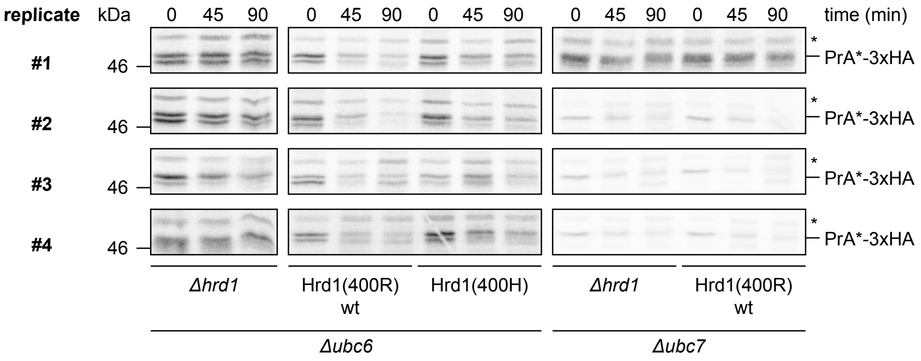

Source Data for Apendix Fig. S1

Protein degradation in indicated yeast strains monitored by pulse-chase experiments for the Hrd1 model substrate PrA*-3xHA. Immunoblots are shown (n = 4), which are the basis for quantifications reported in Apendix Fig. S1. Replicates for the *Δhrd1*, Hrd1(400R) wt, Hrd1(400H), Hrd1(400A), Hrd1(400E), *Δcue1* and *Δcue1*/Hrd1(400R) wt strains as well as all *Δubc6* and *Δdubc7* strains are identical to the ones shown in Source Data for Fig. 3 panel A, Source Data for Fig. 4 panel A and Source Data for Fig. 6 panel A.
