# Supplementary material for: Who with whom: functional coordination of E2 enzymes by RING E3 ligases during poly‐ubiquitylation
Source: EMBO J. 2020 Oct 5;39(22):e104863. doi: 10.15252/embj.2020104863 (PMC7667886; doi:10.15252/embj.2020104863)
Supplement: Supplementary file 5 — Source Data for Figure 2 [file EMBJ-39-e104863-s003.pdf]

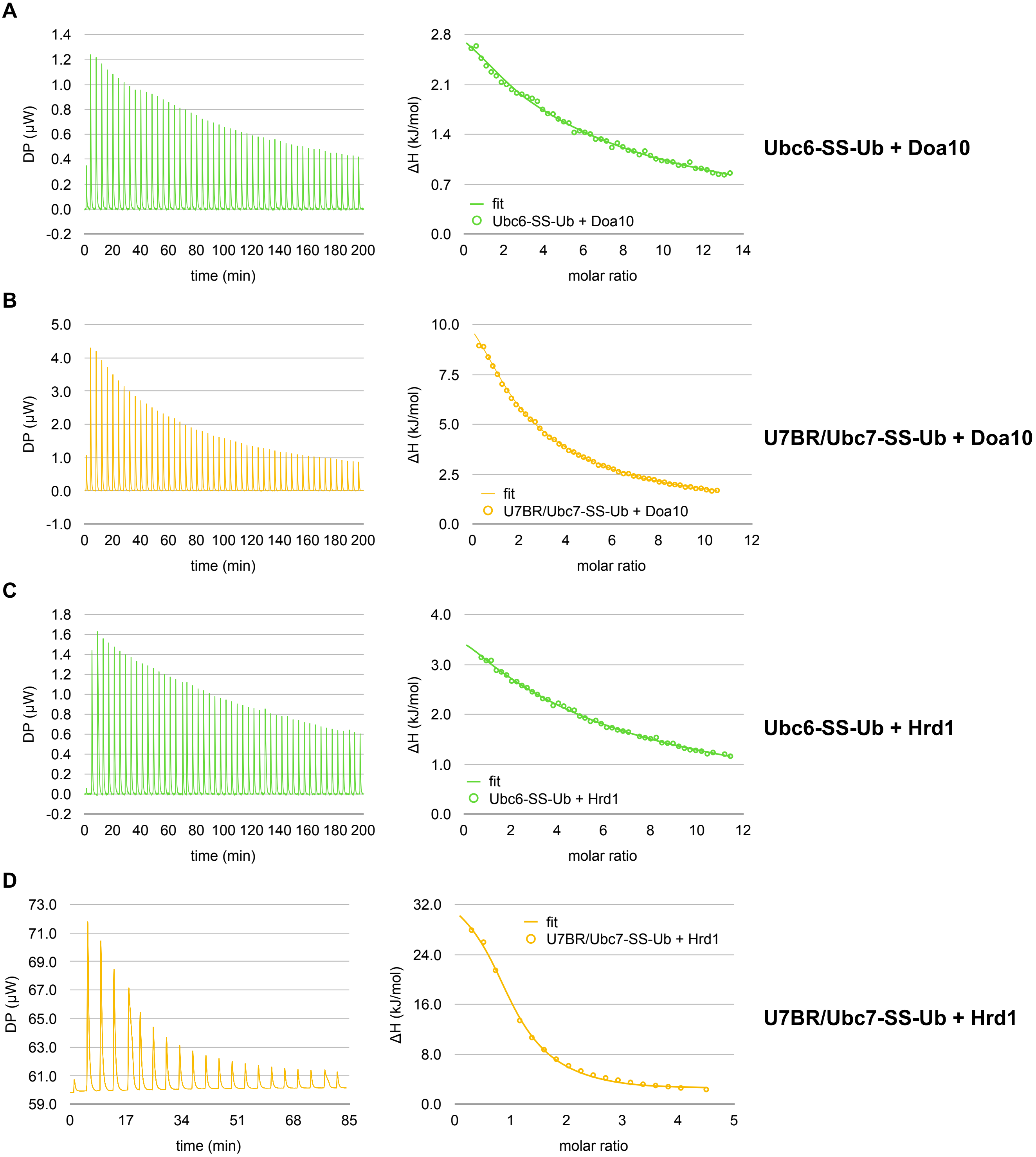
A
DP (μW)
time (min)
ΔH (kJ/mol)
molar ratio
fit
Ubc6-SS-Ub + Doa10
Ubc6-SS-Ub + Doa10
B
DP (μW)
time (min)
ΔH (kJ/mol)
molar ratio
fit
U7BR/Ubc7-SS-Ub + Doa10
U7BR/Ubc7-SS-Ub + Doa10
C
DP (μW)
time (min)
ΔH (kJ/mol)
molar ratio
fit
Ubc6-SS-Ub + Hrd1
Ubc6-SS-Ub + Hrd1
D
DP (μW)
time (min)
ΔH (kJ/mol)
molar ratio
fit
U7BR/Ubc7-SS-Ub + Hrd1
U7BR/Ubc7-SS-Ub + Hrd1

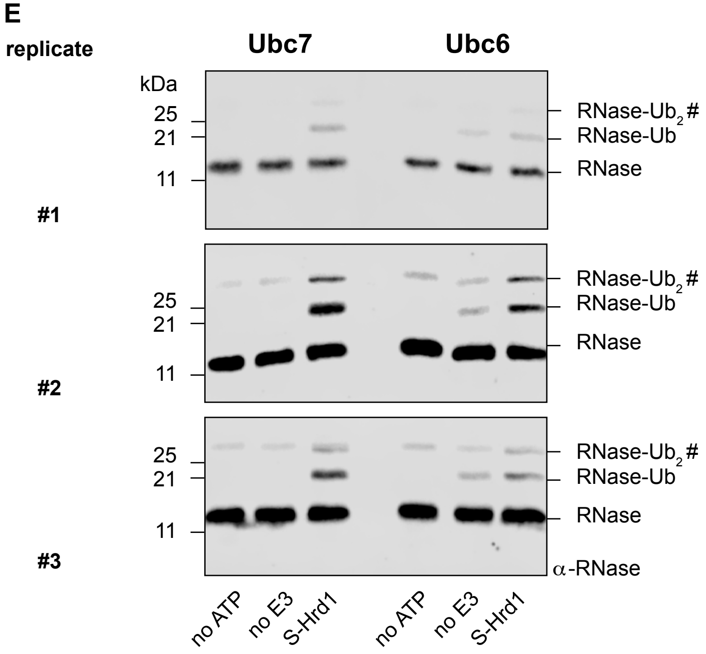

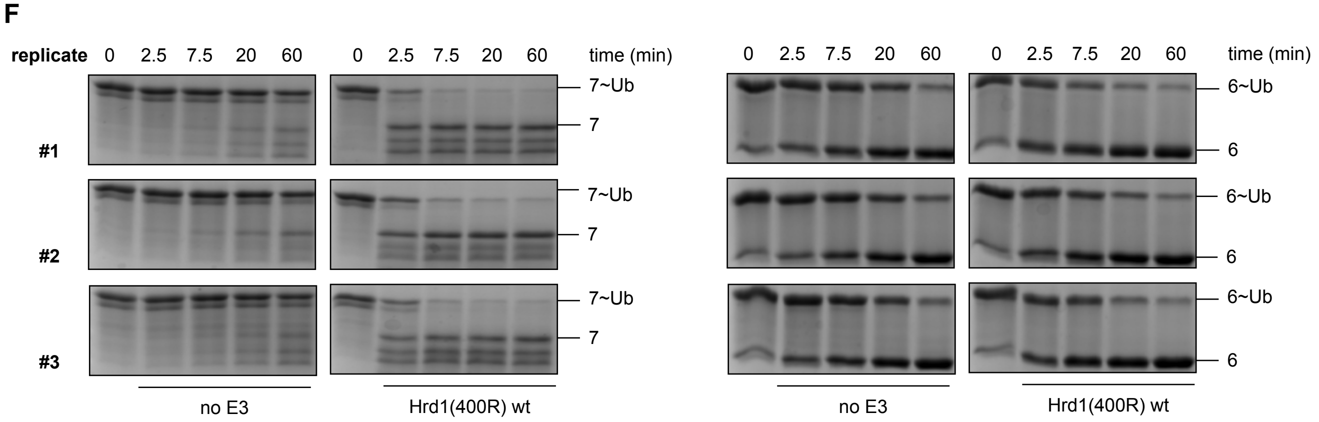

Source Data for Fig. 2

A – D Characterization of E2-SS-Ub/RING interactions by ITC. Raw ITC data (left of each panel) and binding isotherm (right of each panel) of interactions for indicated, disulfide linked E2-SS-Ub conjugates (ITC cell content) with indicated RING domains (titrant) are shown. Due to the weak binding affinities, it was not technically possible to achieve full saturation with attainable protein concentrations in some cases. This data is the basis for K_D values reported in Fig. 2A.

E *In vitro* substrate ubiquitylation assay for Hrd1 with Ubc7 and Ubc6 (A). Immunoblots using a poly-clonal α-RNase A antibody are shown (n = 3). Ubc7 reactions contained equimolar amounts of Cue1 and were performed with Ub(K48R); “no E3” reactions do not contain wild-type S-Hrd1, “no ATP” reactions do not contain ATP, but wild-type S-Hrd1. The RNase-Ub_2 band co-migrates with a nonspecif-ic band (#) common to all samples. Data shown here is the basis for quantifications reported in Fig. 2B. Immunoblots from the frist replicate are shown in the main figures.

F *In vitro* Ub nucleophile discharge assays with U7BR/Ubc7 (left) and Ubc6 (right). Coomassie-stained SDS-PAGE gels under non-reducing conditions are shown (n = 3). Reactions contain ethanolamine as nucleophile and indicated RING variants. “0” time points were taken from the same sample; 6 = Ubc6, 7= Ubc7. Gels shown here are the basis for quantifications and derived rates and fold stimulation reported in Fig. 2D and E, respectively. Representative gels shown in Fig. 2B were not used as replicates for quantifiaction.
