# Supplementary material for: Who with whom: functional coordination of E2 enzymes by RING E3 ligases during poly‐ubiquitylation
Source: EMBO J. 2020 Oct 5;39(22):e104863. doi: 10.15252/embj.2020104863 (PMC7667886; doi:10.15252/embj.2020104863)
Supplement: Supplementary file 6 — Source Data for Figure 3 [file EMBJ-39-e104863-s004.pdf]

A

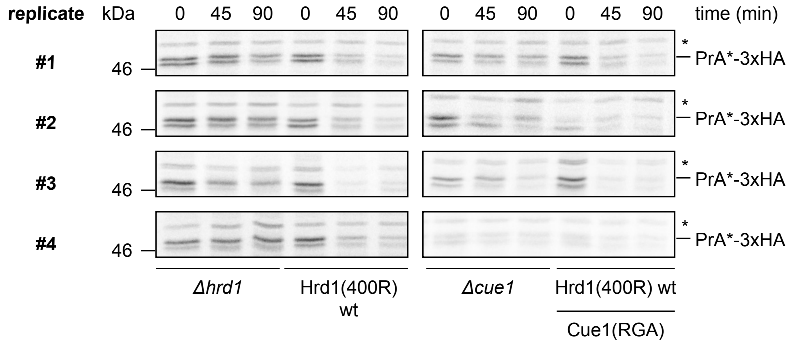

B

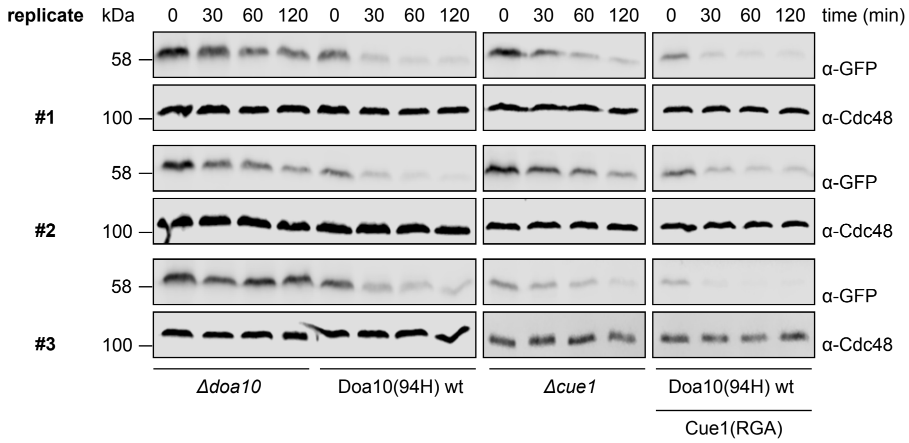

Source Data for Fig. 3

A Protein degradation in indicated yeast strains monitored by pulse-chase experiments for the Hrd1 model substrate PrA*-3xHA. Immunoblots are shown (n = 4), which are the basis for quantifications reported in Fig. 3D - left panel.

B Protein degradation in indicated yeast strains monitored by CHX decay assays for the Doa10 model substrate Deg1-eGFP$_2$. Immunoblots are shown (n = 3), which are the basis for quantifications reported in Fig. 3D - right panel.

The Source Data for Fig. 3A can be found in Fig. EV2 and the Source Data for Fig. EV2.
The Source Data for Fig. 3B can be found in Fig. EV3 and the Source Data for Fig. EV3.
