# Supplementary material for: Who with whom: functional coordination of E2 enzymes by RING E3 ligases during poly‐ubiquitylation
Source: EMBO J. 2020 Oct 5;39(22):e104863. doi: 10.15252/embj.2020104863 (PMC7667886; doi:10.15252/embj.2020104863)
Supplement: Supplementary file 7 — Source Data for Figure 4 [file EMBJ-39-e104863-s005.pdf]

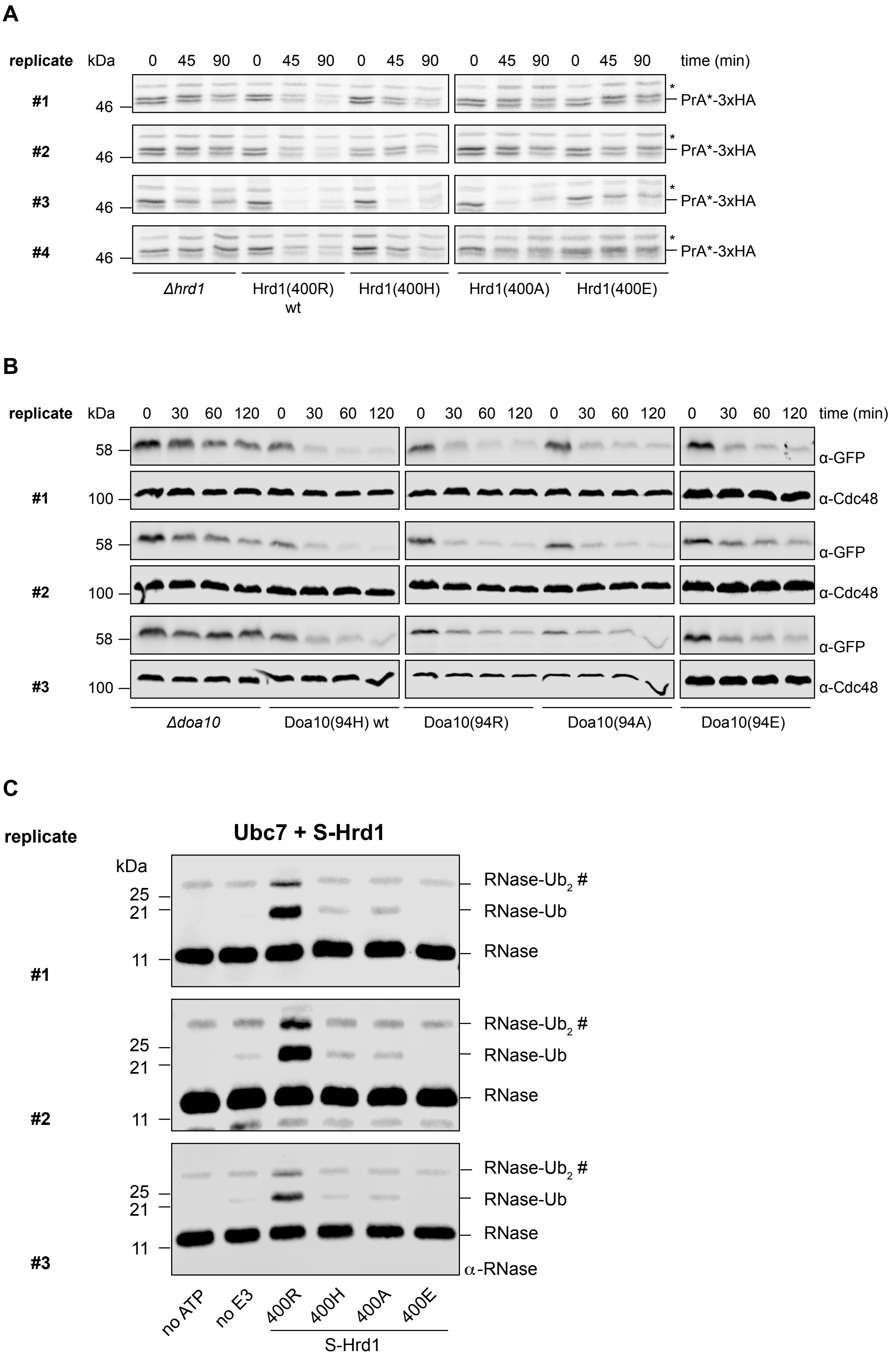
A
replicate
kDa
0 45 90 0 45 90 0 45 90 0 45 90 0 45 90
time (min)
#1
#2
#3
#4
46
*
PrA*-3xHA
Δhrd1
Hrd1(400R) wt
Hrd1(400H)
Hrd1(400A)
Hrd1(400E)
B
replicate
kDa
0 30 60 120 0 30 60 120 0 30 60 120 0 30 60 120 0 30 60 120
time (min)
58
100
α-GFP
α-Cdc48
#1
#2
#3
Δdoa10
Doa10(94H) wt
Doa10(94R)
Doa10(94A)
Doa10(94E)
C
replicate
Ubc7 + S-Hrd1
kDa
25
21
11
RNase-Ub2 #
RNase-Ub
RNase
#1
#2
#3
α-RNase
no ATP
no E3
400R
400H
400A
400E
S-Hrd1

D

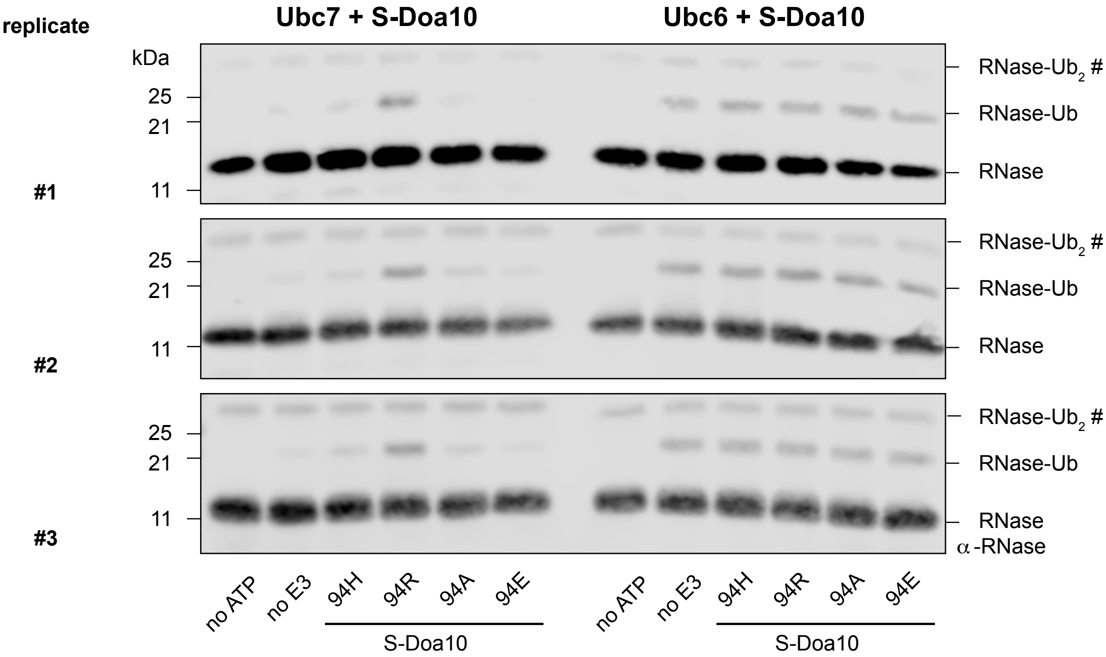

Source Data for Fig. 4

A Protein degradation in indicated yeast strains monitored by pulse-chase experiments for the Hrd1 model substrate PrA*-3xHA. Immunoblots are shown (n = 4), which are the basis for quantifications reported in Fig. 4A - left panel. Replicates for the *Δhrd1* and Hrd1(400R) wt strains are identical to the ones shown in Source Data for Fig. 3 panel A.

B Protein degradation in indicated yeast strains monitored by CHX decay assays for the Doa10 model substrate Deg1-eGFP$_2$. Immunoblots are shown (n = 3), which are the basis for quantifications reported in Fig. 4A - right panel. Replicates for the *Δdoa10* and Doa10(94H) wt strains are identical to the ones shown in Source Data for Fig. 3 panel B.

C - D *In vitro* substrate ubiquitylation assay for Ubc7 with Hrd1 variants (C) and Doa10 variants with Ubc7 and Ubc6 (D). Immunoblot using a poly-clonal α-RNase A antibody (n = 3) are shown. Ubc7 reactions contained equimolar amounts of Cue1 and were performed with Ub(K48R); “no E3” reactions do not contain the respective wild-type S-E3, “no ATP” reactions do not contain ATP, but wild-type S-E3. The RNase-Ub$_2$ band co-migrates with a nonspecific band (#) common to all samples. Data shown here is the basis for quantifications reported in Fig. 4B and 4D. Immunoblots from the frist replicate are shown in the main figures.

The Source Data for Fig. 4C can be found in Fig. EV2 and the Source Data for Fig. EV2.
