# Supplementary material for: Who with whom: functional coordination of E2 enzymes by RING E3 ligases during poly‐ubiquitylation
Source: EMBO J. 2020 Oct 5;39(22):e104863. doi: 10.15252/embj.2020104863 (PMC7667886; doi:10.15252/embj.2020104863)
Supplement: Supplementary file 8 — Source Data for Figure 5 [file EMBJ-39-e104863-s006.pdf]

A

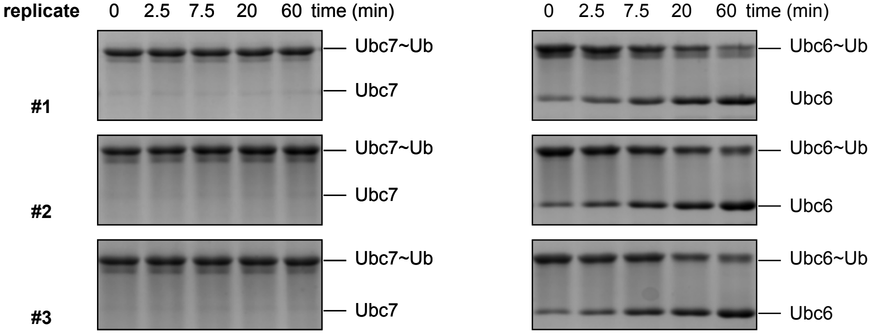

replicate
0
2.5
7.5
20
60
time (min)
Ubc7~Ub
Ubc7
Ubc6~Ub
Ubc6
#1
#2
#3

B

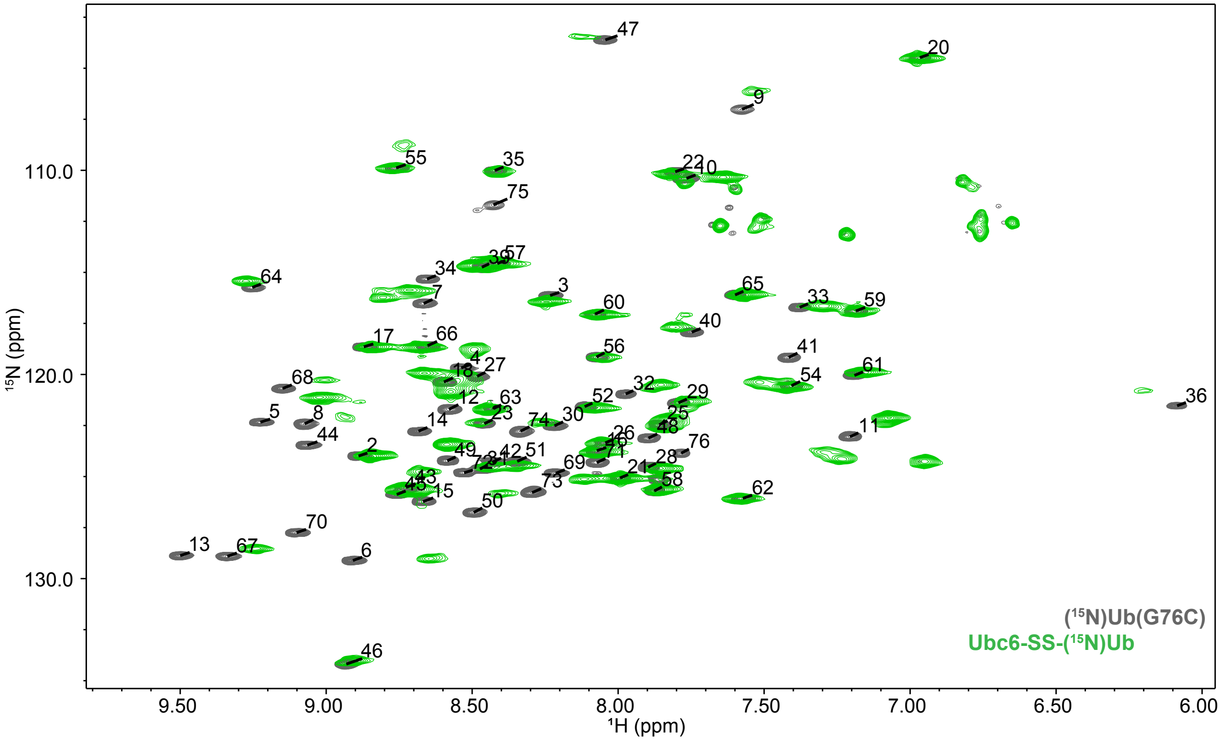

^{15}N (ppm)
^{1}H (ppm)
110.0
120.0
130.0
9.50
9.00
8.50
8.00
7.50
7.00
6.50
6.00
(^{15}N)Ub(G76C)
Ubc6-SS-(^{15}N)Ub

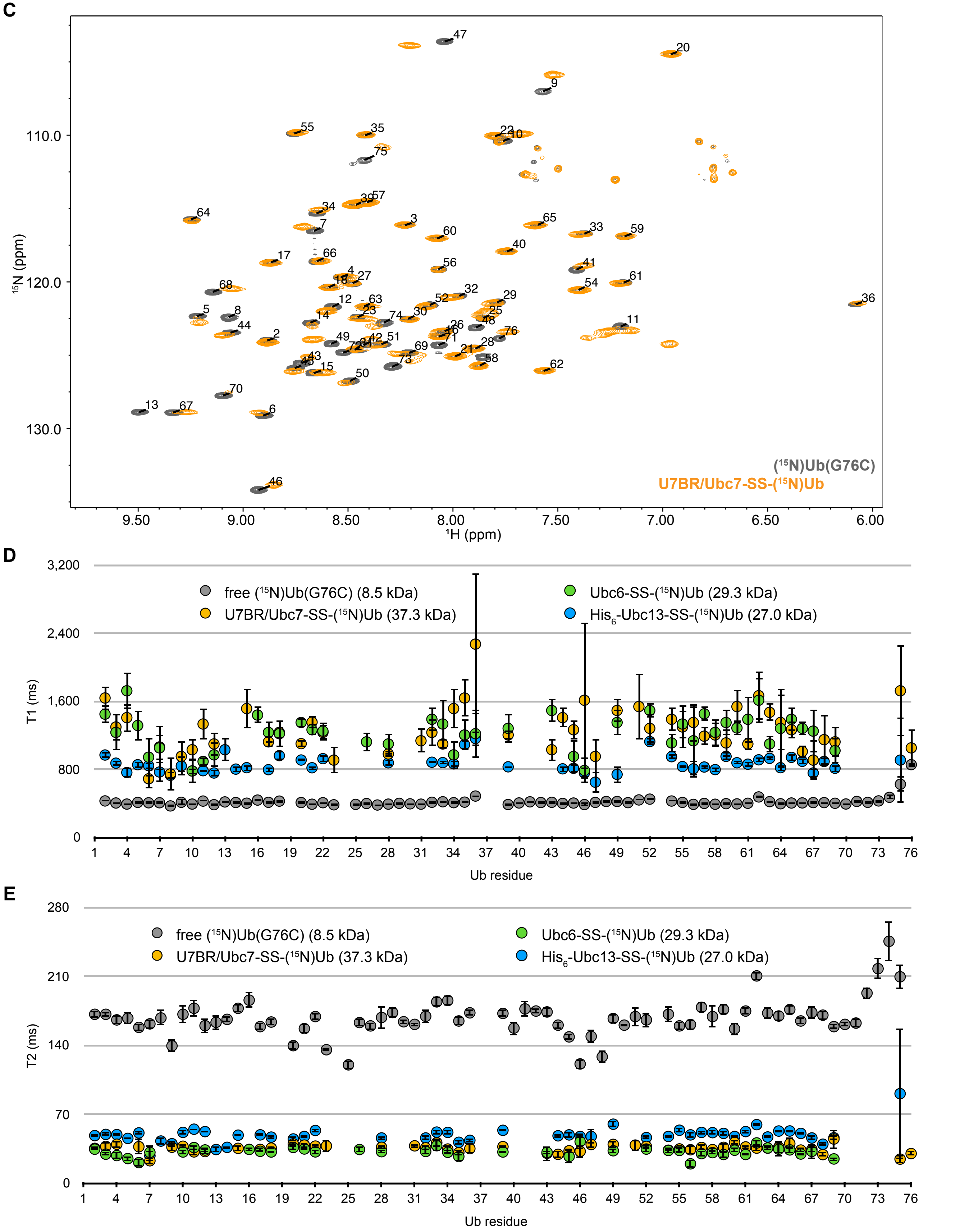
C
(15N)Ub(G76C)
U7BR/Ubc7-SS-(15N)Ub
15N (ppm)
1H (ppm)
D
free (15N)Ub(G76C) (8.5 kDa)
U7BR/Ubc7-SS-(15N)Ub (37.3 kDa)
Ubc6-SS-(15N)Ub (29.3 kDa)
His6-Ubc13-SS-(15N)Ub (27.0 kDa)
T1 (ms)
Ub residue
E
free (15N)Ub(G76C) (8.5 kDa)
U7BR/Ubc7-SS-(15N)Ub (37.3 kDa)
Ubc6-SS-(15N)Ub (29.3 kDa)
His6-Ubc13-SS-(15N)Ub (27.0 kDa)
T2 (ms)
Ub residue

F

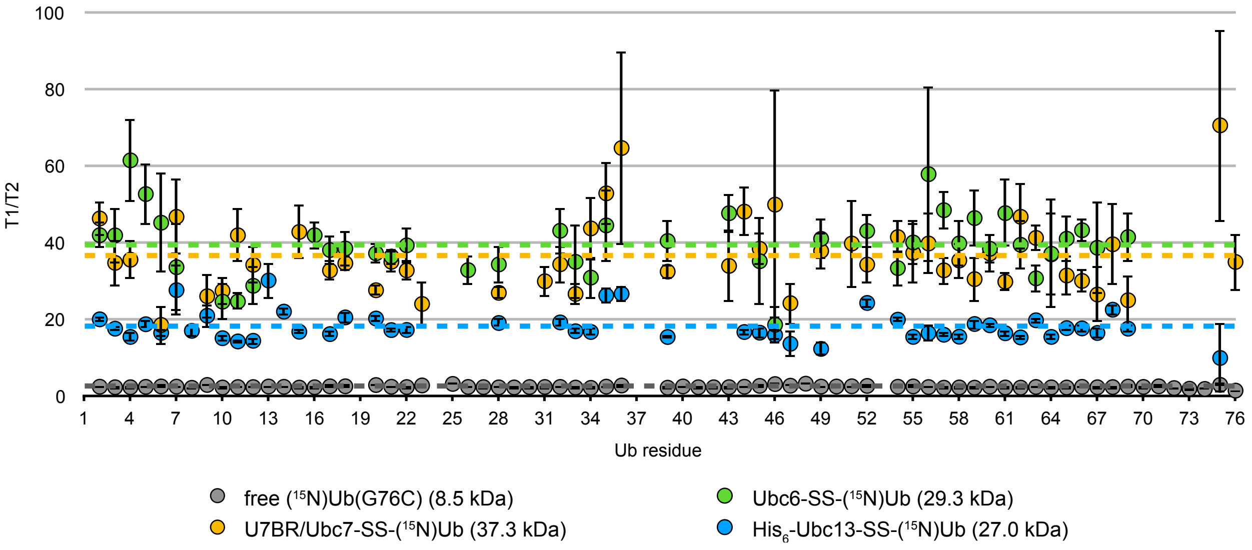

Source Data for Fig. 5

A *In vitro* Ub nucleophile discharge assays with U7BR/Ubc7 (left) and UBc6 (right). Coomassie-stained SDS-PAGE gels under non-reducing conditions are shown (n = 3). Reactions contain no nucleophile (PBS as assays buffer) and were performed at 32°C. Gels shown here are the basis for quantifications reported in Fig. 5A. Representative gels shown in Fig. 5A were not used as replicates for quantifiaction.

B - C Overlaid HSQC-TROSY spectra of free (^{15}N)Ub(G76C) (775 µM - grey spectrum) and of (^{15}N)Ub(G76C) conjugated via disulfide bond to Ubc6 (775 µM - green spectrum in A) and U7BR/Ubc7 (775 µM - yellow spectrum in B). Residue assignments for free Ub(G76C) are shown. For clarity, different contour levels for the spectra of the free and the conjugated Ub are displayed. Spectra are the basis for CSPs reported in Fig. 5B.

D T1 relaxation times determined for residues in (^{15}N)Ub(G76C) conjugates to indicated E2s or in its free form. Residues for which no relaxation time is reported could either not be assigned a peak in the spectrum (see Fig. 5B) or their peaks were of such low intensity or overlapped with other peaks so that a T1 relaxation time analysis was not possible. Errors are reported as the error of each relaxation time based on the fitting process (see Materials & Methods).

E T2 relaxation times determined for residues in (^{15}N)Ub(G76C) conjugates to indicated E2s or in its free form. Residues for which no relaxation time is reported could either not be assigned a peak in the spectrum (see Fig. 5B) or their peaks were of such low intensity or overlapped with other peaks so that a T2 relaxation time analysis was not possible. Errors are reported as the error of each relaxation time based on the fitting process (see Materials & Methods).

F T1/T2 ratios from analyses in D and E for residues in (^{15}N)Ub(G76C) conjugates to indicated E2s or in its free form. Errors were determined by error propagation from the respective errors for each residue shown in A and B. Dotted lines indicate the mean T1/T2 ratio for each construct reported in Fig. 5C.
