# Supplementary material for: Who with whom: functional coordination of E2 enzymes by RING E3 ligases during poly‐ubiquitylation
Source: EMBO J. 2020 Oct 5;39(22):e104863. doi: 10.15252/embj.2020104863 (PMC7667886; doi:10.15252/embj.2020104863)
Supplement: Supplementary file 9 — Source Data for Figure 6 [file EMBJ-39-e104863-s007.pdf]

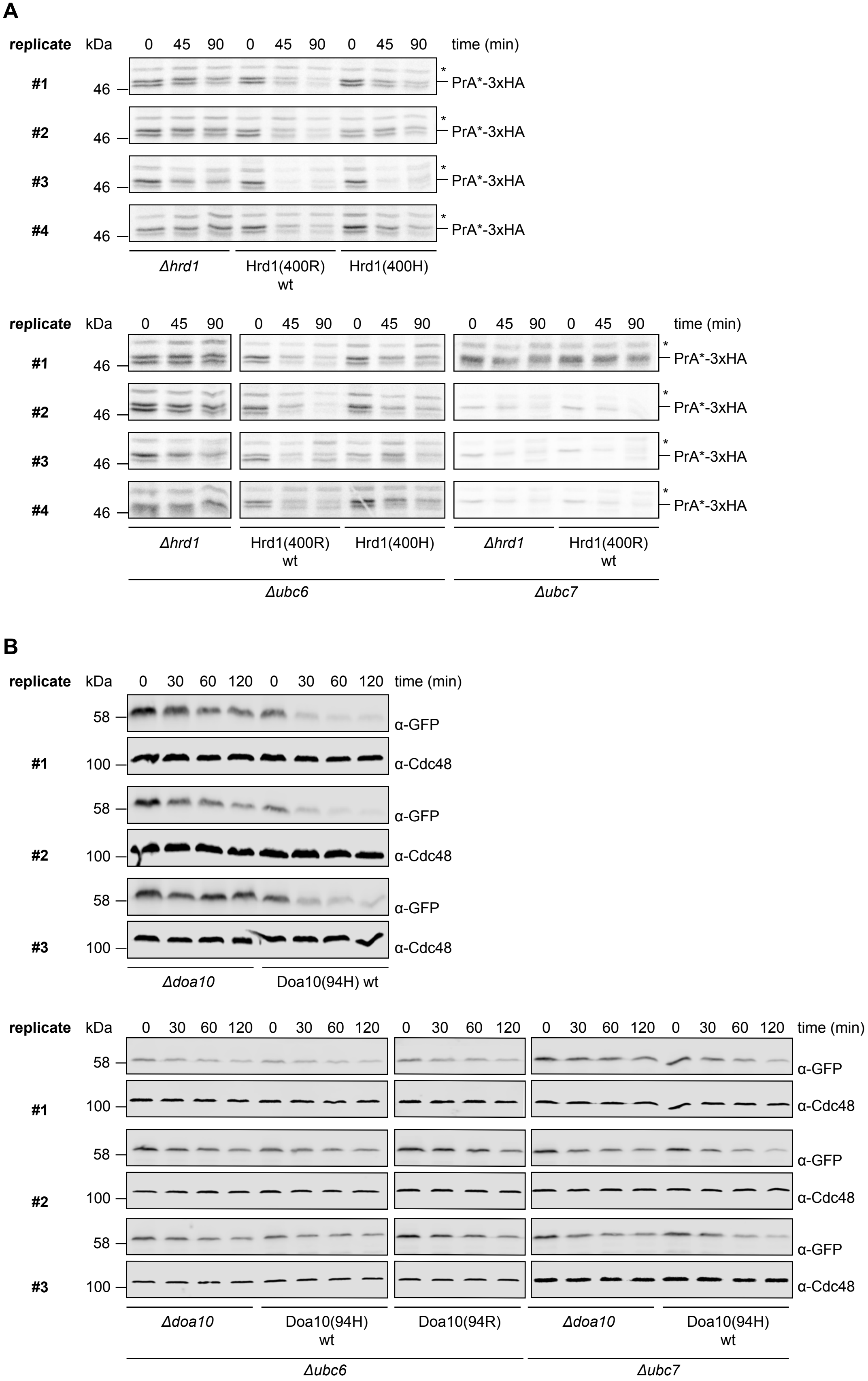
A
replicate
kDa
0 45 90 0 45 90 0 45 90
time (min)
#1
#2
#3
#4
46
*
PrA*-3xHA
Δhrd1
Hrd1(400R) wt
Hrd1(400H)
replicate
kDa
0 45 90 0 45 90 0 45 90 0 45 90 0 45 90
time (min)
Δhrd1
Hrd1(400R) wt
Hrd1(400H)
Δhrd1
Hrd1(400R) wt
Δubc6
Δubc7
B
replicate
kDa
0 30 60 120 0 30 60 120
time (min)
58
100
α-GFP
α-Cdc48
Δdoa10
Doa10(94H) wt
replicate
kDa
0 30 60 120 0 30 60 120 0 30 60 120 0 30 60 120 0 30 60 120
time (min)
Δdoa10
Doa10(94H) wt
Doa10(94R)
Δdoa10
Doa10(94H) wt
Δubc6
Δubc7

C

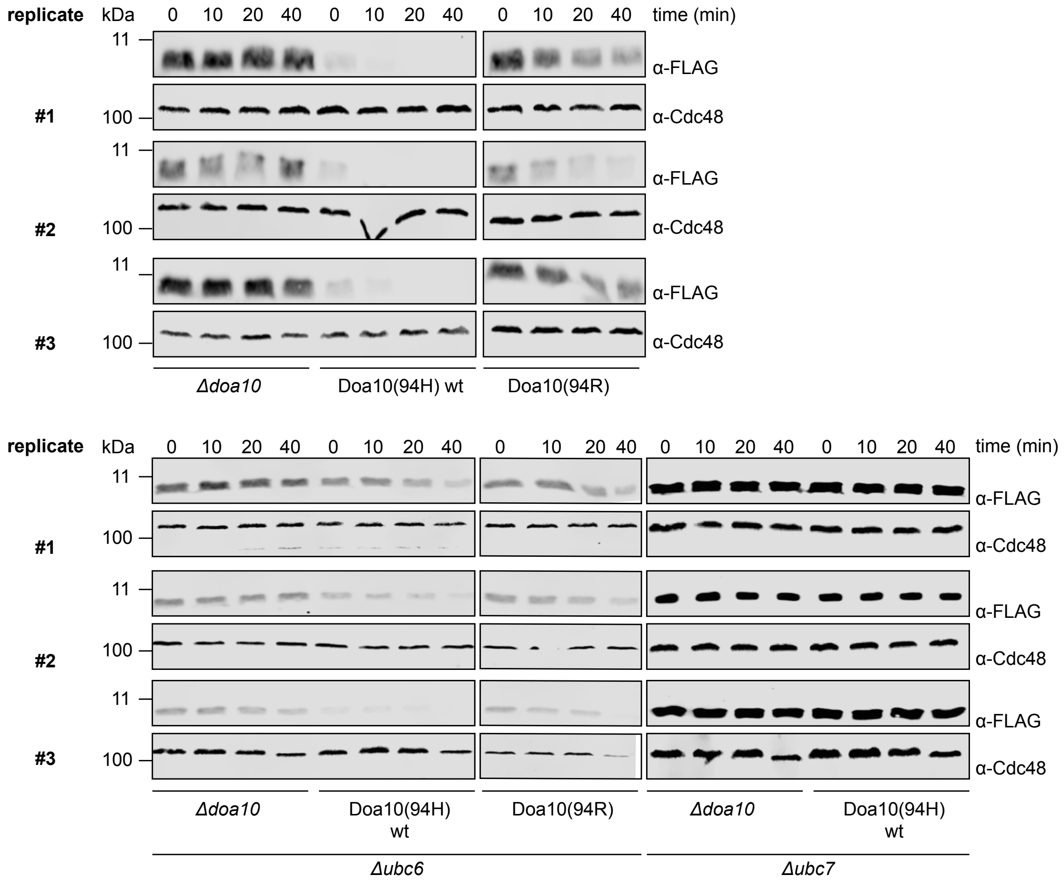

D

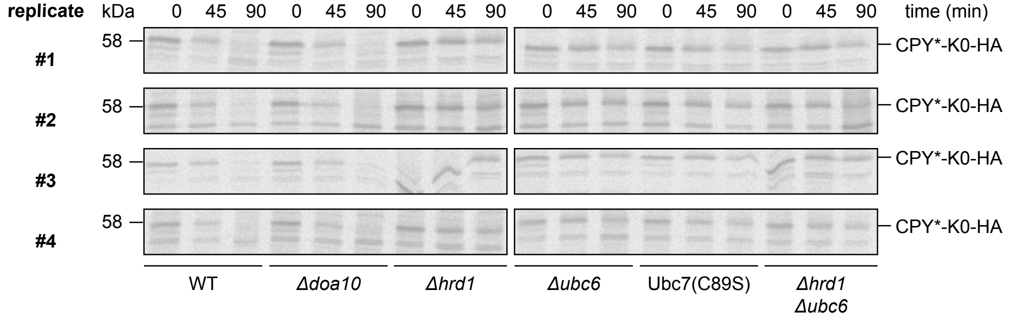

Source Data for Fig. 6

A Protein degradation in indicated yeast strains monitored by pulse-chase experiments for the Hrd1 model substrate PrA*-3xHA. Immunoblots are shown (n = 4), which are the basis for quantifications reported in Fig. 6A and 6D. Replicates for the *Δhrd1*, Hrd1(400R) wt and Hrd1(400H) strains are identical to the ones shown in Source Data for Fig. 3 panel A and Source Data for Fig. 4 panel A.

B Protein degradation in indicated yeast strains monitored by CHX decay assays for the Doa10 model substrate Deg1-eGFP$_2$. Immunoblots are shown (n = 3), which are the basis for quantifications reported in Fig. 6B. Replicates for the *Δdoa10* and Doa10(94H) wt strains are identical to the ones shown in Source Data for Fig. 3 panel B and Source Data for Fig. 4 panel B.

C Protein degradation in indicated yeast strains monitored by CHX decay assays for the Doa10 model substrate FLAG-Sbh2. Immunoblots are shown (n = 3), which are the basis for quantifications reported in Fig. 6C and 6F.

D Protein degradation in indicated yeast strains monitored by pulse-chase experiments for the Hrd1 model substrate CPY*-K0-HA. Immunoblots are shown (n = 4), which are the basis for quantifications reported in Fig. 6E.
